# Epidemiological Evidence for Associations Between Genetic Variants and Osteosarcoma Susceptibility: A Meta-Analysis

**DOI:** 10.3389/fonc.2022.912208

**Published:** 2022-07-04

**Authors:** Dechao Yuan, Jie Tian, Xiang Fang, Yan Xiong, Nishant Banskota, Fuguo Kuang, Wenli Zhang, Hong Duan

**Affiliations:** ^1^ Department of Orthopedics, West China School of Medicine/West China Hospital, Sichuan University, Chengdu, China; ^2^ Department of Thoracic Surgery, West China School of Medicine/West China Hospital, Sichuan University, Chengdu, China; ^3^ Department of Orthopedics, People’s Fourth Hospital of Sichuan Province, Chengdu, China

**Keywords:** meta-analysis, osteosarcoma, single nucleotide polymorphism, susceptibility, Venice criteria

## Abstract

**Background:**

Previous studies have showed that single nucleotide polymorphisms (SNPs) might be implicated in the pathogenesis of osteosarcoma (OS). Numerous studies involving SNPs with OS risk have been reported; these results, however, remain controversial and no comprehensive research synopsis has been performed till now.

**Objective:**

This study seeks to clarify the relationships between SNPs and OS risk using a comprehensive meta-analysis, and assess epidemiological evidence of significant associations.

**Methods:**

The PubMed, Web of Science, and Medline were used to screen for articles that evaluated the association between SNP and OS susceptibility in humans before 24 December 2021. Furthermore, we used Venice Criteria and a false positive report probability (FPRP) test to assess the grades of epidemiological evidence for the statistical relationships.

**Results:**

We extracted useful data based on 43 articles, including 10,255 cases and 13,733 controls. Our results presented that 25 SNPs in 17 genes were significantly associated with OS risk. Finally, we graded strong evidence for 17 SNPs in 14 genes with OS risk (*APE1* rs1760944, *BCAS1* rs3787547, *CTLA4* rs231775, *ERCC3* rs4150506, *HOTAIR* rs7958904, *IL6* rs1800795, *IL8* rs4073, *MTAP* rs7023329 and rs7027989, *PRKCG* rs454006, *RECQL5* rs820196, *TP53* rs1042522, *VEGF* rs3025039, rs699947 and rs2010963, *VMP1* rs1295925, *XRCC3* rs861539), moderate for 14 SNPs in 12 genes and weak for 14 SNPs in 11 genes.

**Conclusion:**

In summary, this study offered a comprehensive meta-analysis between SNPs and OS susceptibility, then evaluated the credibility of statistical relationships, and provided useful information to identify the appropriate candidate SNPs and design future studies to evaluate SNP factors for OS risk.

## Introduction

Osteosarcoma (OS) is one of the most common bone malignancies, occurring mainly in the metaphyseal area around the knee joint (1, 2). Although current treatment strategy, including neoadjuvant therapy prior to wide margin surgical resection and followed by postoperative chemotherapy, greatly improves long-term survival rate to about 70%, its outcome is not satisfactory (3). The pathogenesis of OS is a complex, multistep and multifactorial process in which interactions between genetic and environment factors are proposed to be related to the progression of OS (4, 5). Single nucleotide polymorphisms (SNPs) have been reported to be involved in DNA repair, growth regulation, antigen processing and presentation, which may be implicated in the pathogenesis of OS (6–8). Genetic variants, such as *VEGF* rs2010963, *ERCC* rs1800795, and *IL6* rs1800795, have been found to be related to the susceptibility of lung cancer, gastric cancer, and OS (9–11). Genetic variation plays crucial roles in the pathogenesis of OS and elucidating relationships between genetic variation and OS susceptibility is critical to improve the therapeutic strategies (6, 7).

The study of the association of genetic variation is widely used to filter genes susceptible to OS (6, 7). Although in previous published studies, a single SNP with the risk of OS was investigated, the results were conflicting (9, 12–14). Wang et al. reported that *VEGF* rs3025039 could increase the risk of OS in the recessive model and allelic model (14). However, a study performed by Cao et al. revealed that *VEGF* rs3025039 was not related to OS risk in different genetic models (12). Meta-analysis can assess the consistency of association and increase statistical power, as well as avert repetition and mistakes from previous studies (15). In 2018, Wang et al. summarized the relationships between genetic variants and OS susceptibility only under an allelic model without evaluating cumulative evidence (16). Besides, a comprehensive research synopsis had not been performed to evaluate the epidemiological evidence of genetic relationships between SNP and OS susceptibility till now. To classify cumulative evidence of genetic relationships with OS susceptibility, the Venice Criteria and the false positive report probability (FPRP) test were used in multiple meta-analysis studies (17, 18). Therefore, we aimed to perform an updated meta-analysis to systematically investigate all genetic variation studies of OS risk, then use Venice Criteria and FPRP test to assess the cumulative evidence of the statistical relationships.

## Methods

### Literature Search

**Figure 1:**
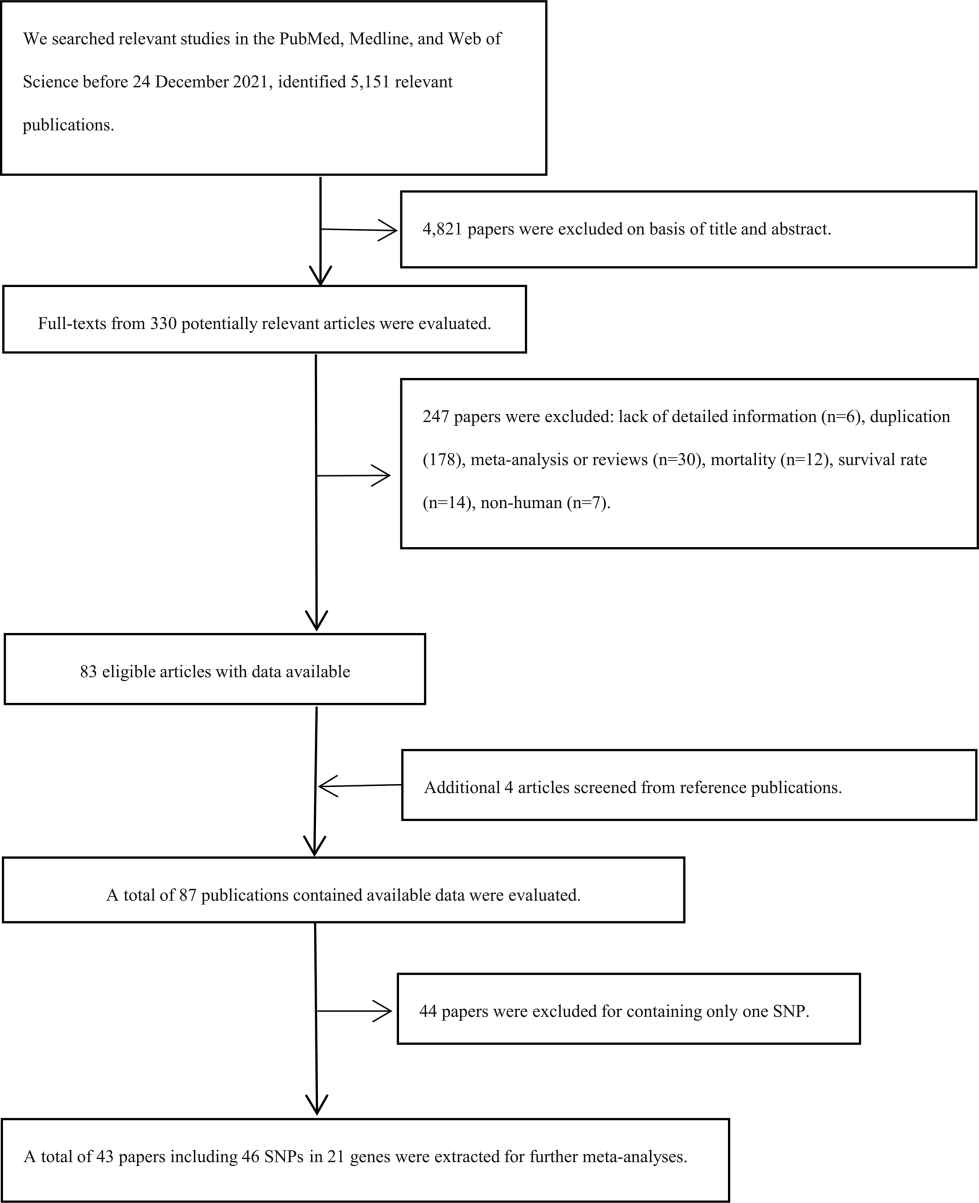
Flow diagram of search strategy and study selection. *SNPs*, single nucleotide polymorphisms.

Preferred Reporting Items for Systematic Reviews and Meta-analysis Statement (PRISMA) were followed in our study (19, 20). We used the PubMed, Web of Science, and Medline to screen the eligible papers before 24 December 2021 using the following terms: ({osteosarcoma} OR {osteogenic sarcoma OR {Sarcoma, Osteogenic}) AND ({variation} OR {variant} OR {single nucleotide polymorphism} OR {polymorphism} OR {SNP}). Moreover, we also screened other relevant articles in the references of the included articles (**Figure 1**).

### Criteria for Inclusion and Exclusion

The included criteria were as follows: (a) evaluation of the association between SNP and OS susceptibility in a case-control in humans; (b) pathologically confirmed OS; (c) providing sufficient information (such as genotype amount); (d) published articles with full text in English. The excluded criteria were as follows: (a) duplicate publications; (b) case reports, reviews, letters, conference abstracts, and meta-analysis; (c) the articles were about the survival/mortality rate of OS.

### Data Extraction

The first author (DY) and co-first author (JT) independently extracted relevant information and then cross-checked each other. If any disagreement was found, discussion and reexamination were made with the third investigator (HD). The following details were extracted: first author, year of publication, variation in gene (rs numbers), gene name, genotype counts, ethnicity, and sample size. Apart from that, three ethnicities (Asian, Mixed and Caucasian) were mentioned in this research; “overall” indicated two or more. Regarding the same SNP with different modes of presentation, we adopted the most recent one on the website (https://www.ncbi.nlm.nih.gov/snp/). The quality of included studies was assessed based on the Newcastle Ottawa Scale (NOS; **Supplementary Table S1**).

### Statistical Analysis

We conducted the study with Stata, version 12 (Stata, College Station, TX, USA), and *P* < 0.05 (two-sided) indicated the significance level in our study. We performed meta-analysis under three models (allelic, dominant, and recessive models) with at least two independent datasets; a subgroup analysis on the basis of ethnicity was also evaluated if necessary. We assessed the heterogeneity of the different studies using the Cochran’s *Q* test and the *I* ^2^ statistic. Specifically, the *I* ^2^ values were assigned at three levels: ≥50%, 25%–50%, ≤25% (21, 22). We used the fixed effect model (*P*_Q_ > 0.1) and the random effect model (*P*_Q_ < 0.1). Furthermore, we conducted sensitivity analysis to evaluate whether significant ORs were lost by excluding the first published study or studies that deviated from the Hardy-Weinberg equilibrium (HWE) in controls. Potential publication bias and small study bias were assessed using Begg’s test and Egger’s test respectively [*P* < 0.1 is the significant level; (23, 24)].

### Evaluation of Cumulative Evidence

The Venice Criteria and FPRP were respectively used to investigate the cumulative epidemiological credibility of significant relationships (17, 18, 25) (**Supporting Information** **for the Venice Criteria and FPRP**). Cumulative epidemiological evidence of statistical relationships was assigned as strong level (all A) or weak level (any C), or moderate level (a combination of A or B) based on Venice Criteria. Ultimately, evidence levels were adjusted according to the FPRP value (cumulative evidence could be downgraded or upgraded according to the FPRP value).

## Results

### Characteristics of the Included Articles

Our research included 5,151 relevant publications, excluded 4,821 papers based on the title and abstract, and excluded 247 papers after a full text review. Apart from that, four papers were screened from reference publications (**Figure 1**). Finally, a total of 43 articles with 46 SNPs in 21 genes were extracted, including 10,255 cases and 13,733 controls (**Supplementary Table S2**). Furthermore, the mean study quality score for included papers was 6.88 ± 0.31 (ranged from 6 to 7) based on NOS (**Supplementary Table S1**).

### Main Meta-Analyses

We conducted a meta-analysis between 46 SNPs in 21 genes and OS risk (a total of 153 associations); of these, 25 SNPs in 17 genes were statistically associated with susceptibility to OS (65 significant associations; **Supplementary Table S3**). Specifically, *APE1* rs1760944, *ERCC3* rs4150506, *HOTAIR* rs7958904, *IL8* rs4073, *MTAP* rs7023329, *MTAP* rs7027989, *PRKCG* rs454006, *RECQL5* rs820196, *VEGF* rs2010963, *VEGF* rs3025039, *VEGF* rs699947, *XRCC1* rs25487 and *XRCC3* rs861539 had significant association with the susceptibility to OS in Asians under three models (allelic model, dominant model, and recessive model). *BCAS1* rs3787547 was significantly associated with susceptibility to OS in Asians (allelic model and dominant model). *CTLA4* rs231775 had statistical relationship with OS susceptibility in overall population and in Asians under three models. For *CTLA4* rs5742909, it was statistically associated with OS susceptibility under recessive model in Asians. We found that *ERCC3* rs4150441 had significant association with OS risk under dominant model in Asians. We found that *HOTAIR* rs874945 was statistically associated with OS susceptibility in Asians under allelic model. *IL10* rs1800896 was significantly associated with risk of OS in overall population (allelic model and dominant model). We found that *IL6* rs1800795 had significant association with OS risk in Asians (allelic model and recessive model). *TNF-α* rs1800629 had statistical association with OS susceptibility in overall population under three models. Our results presented that *TP53* rs1042522 had statistical association with OS susceptibility in overall population (allelic model and dominant model), and in Caucasians (allelic model and dominant model). For *VEGF* rs1570360, our results showed that SNP rs1570360 had statistical association with OS susceptibility in Asians under allelic model. *VEGF* rs833061 had statistical relationship with OS susceptibility in Asians under allelic model. *VMP1* rs1295925 had statistical relationship with OS susceptibility in overall population (allelic model and dominant model; **Table 1**).

**Table 1:** Genetic variants showing significant associations with OS risk in main meta-analyses.

Gene	Variant	Allelic^a^	Ethnicity	Number evaluation		Genetic models	MAF	Effect model	Risk of meta-analysis				Venice Criteria^c^	FPRP values^d^	Credibility of evidence
				Studies	Cases/controls				OR^b^ (95%CI)	I^2^	*P*_Q_	*P*_value_			
*APE1*	rs1760944	T>G	Asian	2	378/616	Allelic	0.442	Fixed	0.692 (0.574–0.834)	0	0.701	<0.001	BAA	0.003	Strong
						Dominant		Fixed	0.610 (0.468–0.796)	0	0.748	<0.001	BAA	0.020	Strong
						Recessive		Fixed	0.642 (0.451–0.914)	0	0.867	0.014	BAA	0.388	Weak
*BCAS1*	rs3787547	G>A	Asian	2	1300/1300	Allelic	0.300	Fixed	1.222 (1.088–1.373)	0	0.703	0.001	AAA	0.014	Strong
						Dominant		Fixed	1.295 (1.110–1.511)	0	0.694	0.001	AAA	0.020	Strong
*CTLA-4*	rs231775	A>G	Overall	4	660/754	Allelic	0.623	Fixed	0.725 (0.620–0.846)	0	0.96	<0.001	AAA	0.001	Strong
						Dominant		Fixed	0.491 (0.360–0.668)	0	0.981	<0.001	AAA	0.004	Strong
						Recessive		Fixed	0.748 (0.596–0.938)	29.9%	0.233	0.012	BBC	0.212	Weak
			Asian	3	594/629	Allelic	0.672	Fixed	0.723 (0.613–0.853)	0	0.862	<0.001	AAA	0.003	Strong
						Dominant		Fixed	0.506 (0.354–0.722)	0	0.97	<0.001	AAA	0.049	Strong
						Recessive		Fixed	0.717 (0.569–0.903)	0	0.711	0.005	BAA	0.109	Moderate
	rs5742909	C>T	Asian	3	486/533	Recessive		Fixed	2.046 (1.028–4.073)	0.0%	0.591	0.042	CAC	0.807	Weak
*ERCC3*	rs4150441	T>C	Asian	2	522/1047	Dominant		Fixed	0.519 (0.357–0.755)	60.1%	0.113	0.001	ACA	0.108	Moderate
	rs4150506	G>A	Asian	2	522/1047	Allelic	0.230	Fixed	1.331 (1.123–1.576)	0	0.581	0.001	BAA	0.019	Strong
						Dominant		Fixed	1.348 (1.089–1.667)	0	0.775	0.006	BAA	0.117	Moderate
						Recessive		Fixed	1.622 (1.110–2.370)	0	0.498	0.012	BAA	0.408	Weak
*HOTAIR*	rs7958904	C>G	Asian	2	900/900	Allelic	0.710	Fixed	1.294 (1.115–1.501)	0	0.736	0.001	AAA	0.013	Strong
						Dominant		Fixed	1.636 (1.154–2.321)	0	0.961	0.006	AAA	0.260	Moderate
						Recessive		Fixed	1.298 (1.078–1.564)	0	0.768	0.006	BAA	0.110	Moderate
	rs874945	C>T	Asian	2	900/900	Allelic	0.189	Fixed	1.183 (1.006–1.393)	17.4%	0.271	0.042	BAA	0.455	Weak
*IL-10*	rs1800896	T>C	Overall	2	340/420	Allelic	0.391	Fixed	1.326 (1.060–1.657)	0	0.557	0.013	BAA	0.224	Weak
						Dominant		Fixed	1.398 (1.009–1.936)	33.9%	0.219	0.044	BBA	0.556	Weak
*IL-6*	rs1800795	C>G	Asian	2	322/322	Allelic	0.750	Random	0.563 (0.445–0.712)	0	0.805	<0.001	BAA	0.000	Strong
						Recessive		Random	0.420 (0.268–0.659)	47.6%	0.167	<0.001	BBA	0.121	Moderate
*IL-8*	rs4073	A>T	Asian	2	299/299	Allelic	0.776	Fixed	0.625 (0.483–0.809)	0	0.793	<0.001	BAA	0.021	Strong
						Dominant		Fixed	0.598 (0.366–0.975)	0	0.949	0.039	BAA	0.692	Weak
						Recessive		Fixed	0.590 (0.424–0.819)	0	0.823	0.002	BAA	0.116	Moderate
*MTAP*	rs7023329	A>G	Asian	2	392/1578	Allelic	0.512	Fixed	0.712 (0.615–0.844)	0	0.540	<0.001	AAA	0.002	Strong
						Dominant		Fixed	0.650 (0.510–0.828)	0	0.439	<0.001	AAA	0.022	Strong
						Recessive		Fixed	0.641 (0.484–0.848)	0	0.855	0.002	BAA	0.082	Moderate
	rs7027989	A>G	Asian	2	392/1578	Allelic	0.824	Fixed	0.761 (0.627–0.923)	0	0.905	0.006	AAA	0.104	Strong
						Recessive		Fixed	0.757 (0.601–0.954)	0	0.760	0.018	AAA	0.288	Moderate
						Dominant		Fixed	0.557 (0.328–0.945)	0	0.751	0.030	AAA	0.693	Moderate
*PRKCG*	rs454006	T>C	Asian	2	998/998	Allelic	0.293	Fixed	1.347 (1.178–1.539)	0	0.826	<0.001	AAA	0.000	Strong
						Dominant		Fixed	1.204 (1.010–1.437)	15.4%	0.277	0.039	AAA	0.432	Moderate
						Recessive		Fixed	1.989 (1.536–2.575)	0	0.596	<0.001	BAA	0.000	Strong
*RECQL5*	rs820196	T>C	Asian	2	397/441	Allelic	0.340	Fixed	1.445 (1.186–1.762)	0	0.742	<0.001	BAA	0.008	Strong
						Dominant		Fixed	1.487 (1.118–1.976)	0	0.844	0.006	BAA	0.184	Moderate
						Recessive		Fixed	2.153 (1.409–3.289)	0	0.700	<0.001	BAA	0.135	Moderate
*TNF-α*	rs1800629	G>A	Overall	2	160/259	Allelic	0.183	Fixed	1.743 (1.245–2.440)	0	0.582	0.001	BAA	0.107	Moderate
						Dominant		Fixed	1.640 (1.065–2.524)	0	0.427	0.025	BAA	0.576	Weak
						Recessive		Fixed	3.306 (1.541–7.093)	0	0.588	0.002	CAA	0.657	Weak
*TP53*	rs1042522	G>C	Overall	3	515/744	Allelic	0.499	Fixed	0.738 (0.618–0.881)	0.0%	0.754	0.001	AAA	0.017	Strong
						Dominant		Fixed	0.591 (0.445–0.784)	14.5%	0.310	<0.001	BAA	0.024	Strong
		G>C	Caucasian	2	305/324	Allelic	0.342	Fixed	0.764 (0.584–0.999)	0.0%	0.503	0.049	BAA	0.526	Weak
						Dominant		Fixed	0.534 (0.364–0.783)	47.4%	0.168	0.001	BBA	0.163	Moderate
*VEGF*	rs1570360	A>G	Asian	3	527/692	Allelic	0.254	Fixed	1.229 (1.025–1.475)	0	0.774	0.026	BAC	0.341	Weak
	rs2010963	C>G	Asian	7	1489/1867	Allelic	0.338	Random	1.249 (1.089–1.432)	46.4%	0.083	0.001	ABA	0.027	Strong
						Dominant		Fixed	1.393 (1.190–1.630)	0	0.504	<0.001	AAA	0.001	Strong
						Recessive		Fixed	1.294 (1.098–1.524)	34.8%	0.163	0.002	BBA	0.038	Strong
	rs3025039	C>T	Asian	8	1671/2049	Allelic	0.230	Fixed	1.248 (1.120–1.391)	0	0.941	<0.001	AAA	0.001	Strong
						Dominant		Fixed	1.222 (1.066–1.399)	0	0.997	0.004	AAC	0.065	Weak
						Recessive		Fixed	1.596 (1.253–2.032)	0	0.702	<0.001	BAA	0.009	Strong
	rs699947	A>C	Asian	4	709/874	Allelic	0.679	Fixed	0.713 (0.615–0.827)	0	0.593	<0.001	AAA	0.000	Strong
						Dominant		Fixed	0.611 (0.462–0.810)	0	0.776	0.001	AAA	0.041	Strong
						Recessive		Fixed	0.685 (0.559–0.840)	0	0.687	<0.001	BAA	0.009	Strong
	rs833061	C>T	Asian	2	358/358	Allelic	0.624	Fixed	0.788 (0.638–0.974)	34.6%	0.216	0.027	BBA	0.358	Weak
*VMP1*	rs1295925	T>C	Asian	2	1300/1300	Allelic	0.450	Fixed	0.847 (0.759–0.945)	0	0.597	0.003	AAA	0.053	Strong
						Dominant		Fixed	0.767 (0.651–0.902)	0	0.646	0.001	AAA	0.026	Strong
*XRCC1*	rs25487	T>C	Asian	2	318/523	Allelic	0.481	Fixed	1.405 (1.132–1.745)	0	0.433	0.002	BAA	0.052	Moderate
						Dominant		Fixed	1.488 (1.055–2.099)	0	0.902	0.024	BAA	0.463	Weak
						Recessive		Fixed	1.564 (1.114–2.195)	50.1%	0.157	0.010	BCA	0.313	Weak
*XRCC3*	rs861539	G>A	Asian	2	288/440	Allelic	0.272	Fixed	1.572 (1.252–1.975)	0	0.882	<0.001	BAA	0.006	Strong
						Dominant		Fixed	1.573 (1.161–2.133)	0	0.902	0.003	BAA	0.151	Moderate
						Recessive		Fixed	2.230 (1.395–3.566)	0	0.896	0.001	CAA	0.240	Weak

APE1, apurinic/apyrimidinic endodeoxyribonuclease 1; BCAS1, brain enriched myelin associated protein 1; CTLA4, cytotoxic T-lymphocyte associated protein 4; ERCC3, excision repair cross-complementation 3; HOTAIR, HOX transcript antisense RNA; IL-10, interleukin-10; IL-6, interleukin-6; IL-8, interleukin-8; MTAP, methylthioadenosine phosphorylase; PRKCG, protein kinase C gamma; RECQL5, RecQ like helicase 5; TNF-α, tumor necrosis factor α; TP53, tumor protein p53; VEGF, vascular endothelial growth factor A; VMP1, vacuole membrane protein 1; XRCC1, X-ray repair cross complementing 1; XRCC3, X-ray repair cross complementing 3; A, adenine; C, cytosine; G, guanine; T, thymine; OR, odds ratio; CI, confidence interval; MAF, minor allelic frequency in control; NA, not applicable; FPRP, false positive report probability.

a Allelic: Minor allelic (bold) versus major allelic (reference).

b OR: OR < 1, decrease the susceptibility of OS (protective factor); OR > 1, increase the susceptibility of OS (susceptive factor).

c Venice Criteria grades are for the amount of evidence, replication of the association, and protection from bias.

d The prior probability of FPRP is 0.05 and the FPRP level of noteworthiness is 0.20.

### Cumulative Evidence of Association

We used the Venice Criteria to assess cumulative epidemiological credibility of significant associations (**Supplementary Table S3**). There were 25 grades A in the amount of evidence, 56 grades A in the replication of association, and 61 grades A in the protection from bias, respectively; there were 37, 7, and 0 grades B in these three criteria, respectively; and there were 3, 2, and 4 grades C were in these three criteria, respectively. The FPRP values were then used to evaluate the significant associations between the 25 SNPs and OS risk (65 associations). 29 associations between 16 SNPs in 14 genes and OS risk obtained a FPRP value < 0.05, as follows: *APE1* rs1760944 (two associations); *BCAS1* rs3787547 (two associations); *CTLA4* rs231775 (four associations); *ERCC3* rs4150506 (one association); *HOTAIR* rs7958904 (one association); *IL6* rs1800795 (one association); *IL8* rs4073 (one association); *MTAP* rs7023329 (two associations); *PRKCG* rs454006 (two associations); *RECQL5* rs820196 (one association); *TP53* rs1042522 (two associations); rs3025039, rs699947, and rs2010963 in *VEGF* (eight associations); *VMP1* rs1295925 (one association); *XRCC3* rs861539 (one association). 16 associations between 15 SNPs in 13 genes and OS risk obtained FPRP 0.05 to 0.2. 20 associations between 16 SNPs in 13 gens and OS risk obtained FPRP value >0.2. Finally, 31 associations with strong evidence were found between 17 SNPs in 14 genes and OS risk (**Table 1**), as follows: *APE1* rs1760944 (two associations); *BCAS1* rs3787547 (two associations); *CTLA4* rs231775 (four associations); *ERCC3* rs4150506 (one association); *HOTAIR* rs7958904 (one association); *IL6* rs1800795 (one association); *IL8* rs4073 (one association); rs7023329, rs7027989 in *MTAP* (three associations); *PRKCG* rs454006 (two associations); *RECQL5* rs820196 (one association); *TP53* rs1042522 (two associations); rs3025039, rs699947, and rs2010963 in *VEGF* (eight associations); *VMP1* rs1295925 (two associations); *XRCC3* rs861539 (one association). 17 associations with moderate evidence were found between 14 SNPs in 12 genes and OS risk; 17 associations with weak evidence were found between 14 SNPs in 11 genes and OS risk.

### Heterogeneity, Bias, and Sensitivity Analysis

56 associations (86.15%) between 23 SNPs in 17 genes and OS risk obtained mild heterogeneity; 7 associations (10.77%) between six SNPs in five genes and OS risk obtained moderate heterogeneity; two associations (3.08%) between two SNPs in two genes and OS risk obtained high heterogeneity (**Table 1**). Publication bias (*p* **<** 0.10 in Begg’s test) was found only in one association (*VEGF* rs3025039 under the dominant model in Asians). After deleting the first published study, the relationships between three SNPs in two genes and OS susceptibility were no longer significant (*CTLA4* rs231775 in the overall population under recessive association; *CTLA4* rs5742909 under recessive association in Asians; *VEGF* rs1570360 under allelic association in Asians; **Supplementary Table S3**).

## Discussion

Our study conducted a comprehensive and updated meta-analysis of the relationships between genetic variants and OS susceptibility. We conducted meta-analysis from 43 papers with 46 SNPs in 21 genes, and found that 25 SNPs in 17 genes were significantly associated with susceptibility to OS (65 significant associations). We further assessed the levels of epidemiological evidence for significant associations combining Venice Criteria as well as FPRP test. Finally, 31 associations with strong epidemiological credibility were found between 17 SNPs in 14 genes and OS risk, as follows: *APE1* rs1760944 (two associations); *BCAS1* rs3787547 (two associations); *CTLA4* rs231775 (four associations); *ERCC3* rs4150506 (one association); *HOTAIR* rs7958904 (one association); *IL6* rs1800795 (one association); *IL8* rs4073 (one association); rs7023329, rs7027989 in *MTAP* (three associations); *PRKCG* rs454006 (two associations); *RECQL5* rs820196 (one association); *TP53* rs1042522 (two associations); rs3025039, rs699947, and rs2010963 in *VEGF* (eight associations); *VMP1* rs1295925 (two associations); *XRCC3* rs861539 (one association).

The apurinic/apyrimidinic endodeoxyribonuclease 1 (*APE1*) gene may be involved in the specific activation of DNA repair and numerous malignancies (26, 27). This study presented strong evidence of the association between a polymorphism (rs1760944) and lower OS risk. SNP rs1760944 (T>G) may impair the binding affinity of octamer-binding transcription factor-1 (*Oct-1*), thus reducing *APE1* mRNA expression levels and then decreasing the risk of OS (28) which is the same as in our meta-analysis. For *APE1* rs1760944, OS patients with G allele had better survival and less susceptible to metastasis, and lower risk of low differentiation tumor (29). The brain enriched myelin associated protein 1 (*BCAS1*) gene resides in a region at 20q13.2 and *BCAS1* rs3787547 may be related to the development of OS by altering the binding power of p53, which is one of the most critical tumor suppressors (30). Our meta-analysis found that *BCAS1* rs3787547 increased the susceptibility of OS with strong evidence in Asians (allelic and dominant model). The excision repair cross-complementation 3 (*ERCC3*) gene encodes a DNA helicase that plays an important role in nucleotide excision repair. The polymorphisms of *ERCC3* have been reported to be associated with several cancers, such as colorectal cancer, pancreatic cancer, breast cancer, and OS (5, 31, 32). The potential mechanism of OS susceptibility was deemed to be its functions as rate-limiting enzymes in the NER pathway (33). Our meta-analysis found that *ERCC3* rs4150506 increased the risk of OS in Asians under the allelic model (strong evidence), dominant model (moderate evidence), and recessive model (weak evidence). The HOX transcript antisense RNA (*HOTAIR*) gene is highly expressed in a variety of cancers, and deletion of HOTAIR can inhibit the aggressiveness of cancers (34). The research by Zhou et al. supported the hypothesis that SNP rs7958904 increased OS risk by influencing lncRNA expression, which was localized to a regulatory boundary in the HOXC cluster (35). Our meta-analysis found that *HOTAIR* rs7958904 increased the risk of OS in Asians (strong evidence in the allelic model, moderate evidence in dominant model, moderate evidence in recessive model), and *HOTAIR* rs874945 increases the risk of OS in Asians under the allelic model with weak evidence.

The interleukin-6 (*IL6*) gene encodes an inflammation cytokine and may be involved in key steps of tumor proliferation, apoptosis, angiogenesis, and differentiation (36). For *IL6* rs1800795, OS patients carrying G allele had better survival and less susceptible to metastasis (10). The interleukin-8 (*IL8*) gene plays a critical role in both the pathogenesis and progression of many human tumors. *IL8* rs4073 is known to affect *IL8* expression that regulates cancer progression through mitogenic and angiogenic factors (37, 38). For IL8 rs4073, OS patients carrying T allele had better Enneking stages and less susceptible to metastasis (39). Our meta-analysis provided strong evidence that *IL6* rs1800795 with the G allele and *IL8* rs4073 with the T allele could decrease the risk of OS under the allelic model in Asians.

The methylthioadenosine phosphorylase (*MTAP*) gene encodes an enzyme that saves methionine and adenine in polyamine metabolism. Inhibition of *MTAP* expression may be responsible for the development of tumor and *MTAP* polymorphisms were associated with some cancer risk, including OS (40, 41). Our meta-analysis also presented strong evidence that *MTAP* rs7023329 (under allelic and dominant model) and rs7027989 (under the allelic model) were associated with a lower risk of OS in Asians. Although the exact mechanism of SNP rs7023329 affect OS risk remains unknown, Zhi et al. hypothesized that SNP rs7023329 might coexist in linkage disequilibrium with one certain variants and affect its regulation machinery to associate with OS risk (42). The protein kinase C gamma (*PRKCG*) gene is located on chromosome 19q13.42 and functions as the major receptor for tumor promoters. Missense variants in exon 4 (C114Y/G123R/G123E) of the *PRKCG* gene have a relationship with tumor development and migration (43). Lu et al. discovered that *PRKCG* rs454006 associated with higher OS risk under allele and dominant model (44). Zheng et al. predicted that SNP rs454006 could cause a new splice donor site, then lead to incorrect translation of the nuclear cancer proteins, which can regulate oncogene products at the transcription level and result in the development of OS (45). Our study found that *PRKCG* rs454006 increased the risk of OS in Asians with strong evidence in the allelic model, moderate evidence in dominant model, and strong evidence in recessive model.

The RecQ like helicase 5 (*RECQL5*) gene is mapped on 17q25.1 and encodes a helicase protein that is essential for genome stability. The *RECQ* family plays a critical role in DNA repair and transcription. Therefore, *RECQL5* variants are considered candidate genes for human cancers (46). As our study found, *RECQL5* rs820196 was associated with higher risk of OS among Asians under allele model (strong evidence). However, the mechanism of how SNP rs820196 affected OS risk has not been revealed. The vacuole membrane protein 1 (*VMP1*) gene encodes a transmembrane protein that plays a key regulatory role in the autophagy process and acts as a tumor suppressors (47, 48). Normal expression of the *VMP1* protein is essential to maintain normal tissue homeostasis and integrity. SNP rs1295925 might affect the binding of p53 and eventually lead to OS susceptibility by affecting the promote or inhibit cell autophagy, and our meta-analysis presented that *VMP1* rs1295925 decreased the risk of OS in Asians with strong evidence [allelic model and dominant model; (49)]. The X-ray repair cross complementing 3 (*XRCC3*) gene encodes a protein that repairs DNA damage and maintains chromosome stability. *XRCC3* polymorphisms influence human cancer susceptibility by altering DNA repair efficiency (50). Our meta-analysis presented that *XRCC3* rs861539 could increase OS susceptibility with strong evidence under the allelic model in Asians. Although the above level of evidence was strong, each SNP only contains 2 datasets with small sample size mainly in Asians, which might reduce the credibility of the results. Therefore, more studies containing a large sample of different ethnicities are needed to evaluated the relationship between OS risk and SNPs above.

The cytotoxic T-lymphocyte associated protein 4 (*CTLA4*) gene encodes a protein that transmits an inhibitory signal to T cells, and plays an important role in increasing cancer susceptibility (51). *CTLA4* rs231775, a variant in which A is changed to G, causes an amino acid exchange and may increase the risk of OS through upregulating the *CTLA4* production and downregulating T cell activation (52, 53). A meta-analysis by Wang et al. revealed that the G allele of SNP rs231775 might function as a protective factor for OS risk (54) which is the same as in our meta-analysis. Our study provided strong evidence that *CTLA4* rs231775 was associated with lower risk of OS (G allele was protective factor) among all populations and Asians both under allelic model and dominant model. However, no significant association was found among Caucasians, which suggesting that more studies were required to evaluate the relationships among Caucasians. The tumor protein p53 (*TP53*) gene is located on chromosome 17p13.1 and acts as a tumor suppressor (55). Savage et al. reported that *TP53* rs1042522 (G > C) increased OS risk under recessive model in a small number of Caucasians (98 cases and 67 controls) (56). However, the association was not significant in our study; instead, our meta-analysis found that *TP53* rs1042522 decreased OS risk in the allelic model (strong evidence) and the dominant model (strong evidence) among overall population, as well as in the allelic model (moderate evidence) and the dominant model (weak evidence) among Caucasians. The decrease in OS risk of SNP rs1042522 may be due to the encoding of a protein isomorph that induces transcription and apoptosis of the target gene (57). The vascular endothelial growth factor A (*VEGF*) gene encodes an angiogenesis cytokine, which induces proliferation and migration of vascular endothelial cells, and the genetic variants of *VEGF* are correlated with tumor risk (58). As our meta-analysis found, *VEGF* rs2010963 increased OS risk with strong evidence under three models among Asians; *VEGF* rs3025039 was associated with higher risk of OS under allelic model and recessive model among Asians (strong evidence); *VEGF* rs699947 decreased OS risk with strong evidence under three models in Asians. Although each SNP mentioned above contains more than two datasets, the sample size is still small, and the population involved is mainly Asian, suggesting that we need to do more research on large populations and different ethnicities in the future. Interestingly, a study performed by Wang et al. (16) revealed that *CTLA4* rs231775, *TP53* rs1042522, *VEGF* rs699947 increased the OS susceptibility in the allelic model, and *VEGF* rs2010963 decreased the OS susceptibility in the allelic model. These results contradicted our meta-analysis because they confused the major allele and the minor allele.

Our research also found that there were 17 relationships between 14 SNPs in 12 genes and OS susceptibility with moderate evidence, and 17 relationships between 14 SNPs in 11 genes and OS susceptibility with weak evidence. Furthermore, large prospective studies should be performed to elucidate the relationships with OS risk for these SNPs with moderate or weak evidence. Additionally, our study that analyzed the same SNP from different groups reported controversial conclusions due to the genetic models, race, and sample size.

Some unavoidable limitations should be noted: (i) although the extensive literature was searched, some papers may have been overlooked; (ii) There could be publication bias because only English articles are examined; (iii) subgroup analysis was conducted based on race (main in Asians and Caucasians) and genetic models, which decrease the credibility of some results; future study in much larger sample size and more races may be needed; and (iv) the errors or confusion of the major allele and the minor allele in the original articles could not be avoided. Therefore, large prospective studies are recommended to evaluate the relationship between OS susceptibility and these SNPs, and all results of our meta-analysis should be interpreted with caution until the molecular properties have been clarified.

Collectively, our research evaluated the cumulative evidence of significant associations of genetic variants with OS risk combining the Venice Criteria and the FPRP test to increase the persuasion and precision of the results. 17 variants in 14 genes with 31 associations were rated as strong evidence of OS susceptibility, 14 SNPs in 12 genes with 17 associations were moderate, and 14 SNPs in 11 genes with 17 associations were weak. Our findings provided useful information to identify the appropriate candidate SNPs and design future studies to evaluate the factors of SNPs for OS risk.

## Data Availability Statement

The original contributions presented in the study are included in the article/**Supplementary Material**. Further inquiries can be directed to the corresponding author.

## Author Contributions

WZ and HD contributed to the conception and design of the study. DY and JT performed the literature search, extracted the data, conducted the meta-analysis, and wrote the first draft of the manuscript. YX, NB, and FK contributed to manuscript editing. XF contributed to the manuscript review. WZ and HD contributed to supervise study, manuscript revision. All authors listed have made a substantial, direct, and intellectual contribution to the work and approved it for publication.

## Conflict of Interest

The authors declare that the research was conducted in the absence of any commercial or financial relationships that could be construed as a potential conflict of interest.

## Publisher’s Note

All claims expressed in this article are solely those of the authors and do not necessarily represent those of their affiliated organizations, or those of the publisher, the editors and the reviewers. Any product that may be evaluated in this article, or claim that may be made by its manufacturer, is not guaranteed or endorsed by the publisher.
