# Supplementary material for: Epidemiological Evidence for Associations Between Genetic Variants and Osteosarcoma Susceptibility: A Meta-Analysis
Source: Front Oncol. 2022 Jul 4;12:912208. doi: 10.3389/fonc.2022.912208 (PMC9291280; doi:10.3389/fonc.2022.912208)
Supplement: Supplementary file 1 [file DataSheet_1.doc]

**Supporting information to Notes** **for the Venice Criteria and the FPRP**

First, we applied the Venice Criteria to evaluate the epidemiological credibility of significant associations identified by meta-analysis. Briefly, credibility was defined as strong, moderate, or weak, based on the grade of A, B, or C in three categories: amount of evidence, replication of the association, and protection from bias(grades A, B, or C were assigned based on each criterion). Cumulative epidemiological evidence of significant associations was rated as a strong association (all criteria grades were A) or weak association (any criteria grades that were C), or moderate association (a combination of A or B).

**Amount of evidence**

A: Large-scale evidence — minor genetic group (alleles or genotypes) in cases and controls > 1,000.

B: Moderate amount of evidence — minor genetic group in cases and controls between 100 and 1,000.

C: Little evidence — minor genetic group in cases and controls < 100.

**Replication of association**

A: Little between-study heterogeneity — *I*^2^ < 25%.

B: Moderate between-study heterogeneity — *I*^2^ between 25% and 50%.

C: Large between-study heterogeneity — *I*^2^ > 50%.

Qualitative epidemiologic considerations about the presence of heterogeneity and potential explanation for heterogeneity would need to be taken into account in judging replication. It may be reasonable to grade as A on this criterion for associations with moderate or high heterogeneity with an extensive replication record such as associations identified by GWAS or large GWAS meta-analysis from collaborative studies.

**Protection from bias**

A: No observable bias and bias was unlikely to explain the presence of the association. B: No obvious bias may affect the presence of the association, but there is considerable missing information on the identification of evidence. C: Bias is demonstrable or is likely to explain the presence of the association. The Venice criteria include an extensive checklist for sources of bias in different settings. The checklist has different considerations depending on whether the evidence comes from retrospective meta-analyses of published data or prospective GWAS and replication studies from collaborative consortia with harmonization of data collection and analysis.

General checks for bias that have been adopted for meta-analysis are: (1) Association lost with exclusion of first study; (2) Association lost with exclusion of studies deviated from HWE; (3) Small effect size of association (i.e., 0.87 < OR < 1.15); (4) Evidence of publication bias (*p* < 0.10 in Begg's test); (5) Evidence of small-study effect (*p* < 0.10 in Egger's test); (6) Evidence is presented for an excess of individual studies with significant findings (*p* < 0.10 in significant bias test).

**FPRP**

A prior probability of 0.05 and an FPRP cut-off value of 0.2 in the FPRP assay should be performed to detect potential false positive results among statistical associations and assess whether these associations should be excluded. If the calculated FPRP value was below the prespecified noteworthiness value of 0.2, we would consider the association noteworthy, indicating the association might be true. The evidence levels were classified according to the FPRP value: < 0.05, 0.05 – 0.2, > 0.2, indicating strong, moderate, or weak, respectively. The cumulative evidence could be upgraded from moderate to strong or from weak to moderate based on a strong FPRP (< 0.05). Otherwise, cumulative evidence could be downgraded from strong to moderate or from moderate to weak based on weak FPRP (> 0.2).

**Supplementary Table S1: Newcastle Ottawa Scale (NOS) of case-control studies was used to evaluate the quality for each eligible study.**

| **Author, year^(Ref)^** | **Selection** | | | | **Comparability** | **EXPOSURE** | | | **Overall quality** |
| --- | --- | --- | --- | --- | --- | --- | --- | --- | --- |
|  | **Is the Case Definition Adequate?** | **Representativeness of**  **the Cases** | **Selection of**  **Controls** | **Definition**  **of**  **Controls** | **Comparability of Cases and Controls on the Basis of the Design or Analysis** | **Ascertainment of**  **Exposure** | **Same method of ascertainment for cases and controls** | **Non-Response Rate** |  |
| Wu Y, 2018 | ★ | ★ | ★ | ★ | ★☆ | ★ | ★ | ☆ | **7** |
| Zhang J I^*^, 2018 | ★ | ★ | ★ | ★ | ★☆ | ★ | ★ | ☆ | **7** |
| Zhang J II^＃^, 2018 | ★ | ★ | ★ | ★ | ★☆ | ★ | ★ | ☆ | **7** |
| Xu Q, 2017 | ★ | ★ | ★ | ★ | ★☆ | ★ | ★ | ☆ | **7** |
| Bilbao-Aldaiturriaga N, 2017 | ★ | ★ | ★ | ★ | ★☆ | ★ | ★ | ☆ | **7** |
| Qiao G, 2017 | ★ | ★ | ★ | ★ | ★☆ | ★ | ★ | ☆ | **7** |
| Niu J, 2017 | ★ | ★ | ★ | ★ | ☆☆ | ★ | ★ | ☆ | **6** |
| Xiao X I^*^, 2017 | ★ | ★ | ★ | ★ | ★☆ | ★ | ★ | ☆ | **7** |
| Xiao X II^＃^, 2017 | ★ | ★ | ★ | ★ | ★☆ | ★ | ★ | ☆ | **7** |
| Zhi L I^*^, 2016 | ★ | ★ | ★ | ★ | ★☆ | ★ | ★ | ☆ | **7** |
| Zhi L II^＃^, 2016 | ★ | ★ | ★ | ★ | ★☆ | ★ | ★ | ☆ | **7** |
| Ma X , 2016 | ★ | ★ | ★ | ★ | ☆☆ | ★ | ★ | ☆ | **6** |
| Zhou Q I^*^, 2016 | ★ | ★ | ★ | ★ | ★☆ | ★ | ★ | ☆ | **7** |
| Zhou Q II^＃^, 2016 | ★ | ★ | ★ | ★ | ★☆ | ★ | ★ | ☆ | **7** |
| Qi Y, 2016 | ★ | ★ | ★ | ★ | ★☆ | ★ | ★ | ☆ | **7** |
| Chen Y, 2016 | ★ | ★ | ★ | ★ | ★☆ | ★ | ★ | ☆ | **7** |
| Cui Y, 2016 | ★ | ★ | ★ | ★ | ☆☆ | ★ | ★ | ☆ | **6** |
| Wang K, 2016 | ★ | ★ | ★ | ★ | ★☆ | ★ | ★ | ☆ | **7** |
| Xu T, 2016 | ★ | ★ | ★ | ★ | ★☆ | ★ | ★ | ☆ | **7** |
| Wang Z, 2016 | ★ | ★ | ★ | ★ | ★☆ | ★ | ★ | ☆ | **7** |
| Mei J, 2016 | ★ | ★ | ★ | ★ | ★☆ | ★ | ★ | ☆ | **7** |
| Cao L, 2016 | ★ | ★ | ★ | ★ | ★☆ | ★ | ★ | ☆ | **7** |
| Guo J, 2015 | ★ | ★ | ★ | ★ | ★☆ | ★ | ★ | ☆ | **7** |
| Liu J, 2015 | ★ | ★ | ★ | ★ | ★☆ | ★ | ★ | ☆ | **7** |
| Yang L, 2015 | ★ | ★ | ★ | ★ | ★☆ | ★ | ★ | ☆ | **7** |
| Hu G, 2015 | ★ | ★ | ★ | ★ | ★☆ | ★ | ★ | ☆ | **7** |
| Zhang H, 2015 | ★ | ★ | ★ | ★ | ★☆ | ★ | ★ | ☆ | **7** |
| Zhang G, 2015 | ★ | ★ | ★ | ★ | ★☆ | ★ | ★ | ☆ | **7** |
| Zhao L, 2015 | ★ | ★ | ★ | ★ | ★☆ | ★ | ★ | ☆ | **7** |
| Zhao Z, 2015 | ★ | ★ | ★ | ★ | ★☆ | ★ | ★ | ☆ | **7** |
| Ru J, 2015 | ★ | ★ | ★ | ★ | ☆☆ | ★ | ★ | ☆ | **6** |
| Dong Y, 2015 | ★ | ★ | ★ | ★ | ★☆ | ★ | ★ | ☆ | **7** |
| Gómez-Díaz B, 2015 | ★ | ★ | ★ | ★ | ★☆ | ★ | ★ | ☆ | **7** |
| Lu H, 2015 | ★ | ★ | ★ | ★ | ★☆ | ★ | ★ | ☆ | **7** |
| Wu Y, 2015 | ★ | ★ | ★ | ★ | ★☆ | ★ | ★ | ☆ | **7** |
| Tie Z, 2014 | ★ | ★ | ★ | ★ | ★☆ | ★ | ★ | ☆ | **7** |
| Zhang Y, 2014 | ★ | ★ | ★ | ★ | ★☆ | ★ | ★ | ☆ | **7** |
| Jiang C, 2014 | ★ | ★ | ★ | ★ | ★☆ | ★ | ★ | ☆ | **7** |
| Wang Z, 2014 | ★ | ★ | ★ | ★ | ★☆ | ★ | ★ | ☆ | **7** |
| Zhi L, 2014 | ★ | ★ | ★ | ★ | ★☆ | ★ | ★ | ☆ | **7** |
| Xu S, 2014 | ★ | ★ | ★ | ★ | ★☆ | ★ | ★ | ☆ | **7** |
| Liu Y, 2011 | ★ | ★ | ★ | ★ | ★☆ | ★ | ★ | ☆ | **7** |
| Wang W, 2011 | ★ | ★ | ★ | ★ | ☆☆ | ★ | ★ | ☆ | **6** |
| Biason P, 2011 | ★ | ★ | ★ | ★ | ★☆ | ★ | ★ | ☆ | **7** |
| Toffoli G, 2009 | ★ | ★ | ★ | ★ | ★☆ | ★ | ★ | ☆ | **7** |
| Oliveira I, 2007 | ★ | ★ | ★ | ★ | ★☆ | ★ | ★ | ☆ | **7** |
| Savage S, 2007 | ★ | ★ | ★ | ★ | ★☆ | ★ | ★ | ☆ | **7** |

Note: I^*^, stage I; II^＃^, stage II.

No.of studies: **43**

Overall quality of all study: 296

**Average: 6.88**

References:

[1] Wu YG, Li HF, Ren YJ, Zou DB, Zhang KN, Xiao X. The association of XRCC1 polymorphism with osteosarcoma risk, clinicopathologic features, and prognosis in a China Han population [J]. Cancer Manag Res, 2018, 10: 4959-67.

[2] Zhang J, Kai L, Zhang W, Yin Y, Wang W. Association between genetic variants in p53 binding sites and risks of osteosarcoma in a China population: a two-stage case-control study [J]. Cancer Biol Ther, 2018, 19(11): 994-7.

[3] Xu Q, Zhang Z, Sun W, Hu B. Haplotype analysis on relationship of ERCC2 and ERCC3 gene polymorphisms with osteosarcoma risk in China young population [J]. Mamm Genome, 2017, 28(5-6): 227-33.

[4] Bilbao-Aldaiturriaga N, Patino-Garcia A, Martin-Guerrero I, Garcia-Orad A. Cytotoxic T lymphocyte-associated antigen 4 rs231775 polymorphism and osteosarcoma [J]. Neoplasma, 2017, 64(2): 299-304.

[5] Qiao G, Miao H, Yi Y, Wang D, Liu B, Zhang Y, et al. Genetic association between CTLA4 variations and osteosarcoma risk: case-control study [J]. International Journal of Clinical and Experimental Medicine, 2016, 9(6): 9598-602.

[6] Niu J, Zhao X, Liu Q, Liu G. Interleukin-6 gene-174 G/C polymorphis mare correlated with susceptibility of osteosarcoma in China population [J]. International Journal of Clinical and Experimental Medicine, 2017, 10(8): 12595-9.

[7] Xiao X, Yang Y, Ren Y, Zou D, Zhang K, Wu Y. rs1760944 Polymorphism in the APE1 Region is Associated with Risk and Prognosis of Osteosarcoma in the China Han Population [J]. Sci Rep, 2017, 7(1): 9331.

[8] Zhi L, Liu D, Wu SG, Li T, Zhao G, Zhao B, et al. Association of common variants in MTAP with susceptibility and overall survival of osteosarcoma: a two-stage population-based study in Han China [J]. J Cancer, 2016, 7(15): 2179-86.

[9] Ma X, Zhang Y, Sun TS, Yao JH. Role of ERCC2 and ERCC3 gene polymorphisms in the development of osteosarcoma [J]. Genet Mol Res, 2016, 15(1).

[10] Zhou Q, Chen F, Fei Z, Zhao J, Liang Y, Pan W, et al. Genetic variants of lncRNA HOTAIR contribute to the risk of osteosarcoma [J]. Oncotarget, 2016, 7(15): 19928-34.

[11] Qi Y, Zhao C, Li H, Zhang B, Tada K, Abe H, et al. Genetic variations in interleukin-6 polymorphism and the association with susceptibility and overall survival of osteosarcoma [J]. Tumour Biol, 2016, 37(7): 9807-11.

[12] Chen Y, Yang Y, Liu S, Zhu S, Jiang H, Ding J. Association between interleukin 8 -251 A/T and +781 C/T polymorphisms and osteosarcoma risk in China population: a case-control study [J]. Tumour Biol, 2016, 37(5): 6191-6.

[13] Cui Y, Zhu JJ, Ma CB, Cui K, Wang F, Ni SH, et al. Interleukin 10 gene -1082A/G polymorphism is associated with osteosarcoma risk and poor outcomes in the China population [J]. Tumour Biol, 2016, 37(4): 4517-22.

[14] Wang K, Zhao J, He M, Fowdur M, Jiang T, Luo S. Association of GRM4 gene polymorphisms with susceptibility and clinicopathological characteristics of osteosarcoma in Guangxi China population [J]. Tumour Biol, 2016, 37(1): 1105-12.

[15] Tian X, Chen YY, Fang GW, Zhou ZG, Dong JM, Guo Q. Association between IL8 rs4073 polymorphisms and osteosarcoma risk in China population: a case control study [J]. International Journal of Clinical and Experimental Medicine, 2016, 9(7): 13172-7.

[16] Wang Z, Wu N. Association between XRCC1 and ERCC2 gene polymorphisms and development of osteosarcoma [J]. International Journal of Clinical and Experimental Pathology, 2016, 9(1): 223-9.

[17] Mei J-W, Huang R, Gao F, Sun H-g. Association between CTLA4 polymorphisms and osteosarcoma susceptibility [J]. International Journal of Clinical and Experimental Pathology, 2016, 9(2): 2265-70.

[18] Cao L, Zhang S, Ma W. The vascular endothelial growth factor (VEGF) gene rs2010963 and rs3025039 polymorphisms and risk of osteosarcoma in China population: evidence from a case-control study and a meta-analysis [J]. International Journal of Clinical and Experimental Pathology, 2016, 9(11): 11276-88.

[19] Guo J, Lv HC, Shi RH, Liu WL. Association between XRCC3 Thr241Met polymorphism and risk of osteosarcoma in a China population [J]. Genet Mol Res, 2015, 14(4): 16484-90.

[20] Liu JQ, Bai X, Duan DC, Dou AX. Role of five small nucleotide polymorphisms in the VEGF gene on the susceptibility to osteosarcoma and overall survival of patients [J]. Oncol Lett, 2015, 10(3): 1481-6.

[21] Yang L, An Y, Wang G, Lu T, Yang S. Association between XRCC3 Thr241Met polymorphism and risk of osteosarcoma in a China population [J]. Int J Clin Exp Pathol, 2015, 8(9): 11670-4.

[22] Hu GL, Ma G, Ming JH. Impact of common SNPs in VEGF gene on the susceptibility of osteosarcoma [J]. Genet Mol Res, 2015, 14(4): 14561-6.

[23] Zhang HF, Yan JP, Zhuang YS, Han GQ. Association between angiogenic growth factor genetic polymorphisms and the risk of osteosarcoma [J]. Genet Mol Res, 2015, 14(3): 10524-9.

[24] Zhang G, Bai R, Zhang T, Zhang H, Wen SZ, Jiang DM. Investigation of the role of VEGF gene polymorphisms in the risk of osteosarcoma [J]. Genet Mol Res, 2015, 14(3): 8283-9.

[25] Zhao L, Wang L, Shi L, Zhang Y. Investigation on the role of VEGF gene polymorphisms in the risk of osteosarcoma [J]. Pak J Med Sci, 2015, 31(2): 364-8.

[26] Zhao Z, Tang X, Song K, Li X, Zhang Y. Association of -308G/A and -238G/A polymorphisms of TNF-α and osteosarcoma risk [J]. Int J Clin Exp Pathol, 2015, 8(4): 4177-81.

[27] Ru JY, Cong Y, Kang WB, Yu L, Guo T, Zhao JN. Polymorphisms in TP53 are associated with risk and survival of osteosarcoma in a China population [J]. Int J Clin Exp Pathol, 2015, 8(3): 3198-203.

[28] Dong YZ, Huang YX, Lu T. Single nucleotide polymorphism in the RECQL5 gene increased osteosarcoma susceptibility in a China Han population [J]. Genet Mol Res, 2015, 14(1): 1899-902.

[29] Gómez-Díaz B, M DLLA-M, Gutiérrez-Angulo M, Valle-Solis AE, Linares-González LM, González-Guzmán R, et al. Analysis of ERCC1 and ERCC2 gene variants in osteosarcoma, colorectal and breast cancer [J]. Oncol Lett, 2015, 9(4): 1657-61.

[30] Lu H, Zhu L, Lian L, Chen M, Shi D, Wang K. Genetic variations in the PRKCG gene and osteosarcoma risk in a China population: a case-control study [J]. Tumour Biol, 2015, 36(7): 5241-7.

[31] Wu Y, Zhao J, He M. Correlation between TGF-β1 gene 29 T > C single nucleotide polymorphism and clinicopathological characteristics of osteosarcoma [J]. Tumour Biol, 2015, 36(7): 5149-56.

[32] Tie Z, Bai R, Zhai Z, Zhang G, Zhang H, Zhao Z, et al. Single nucleotide polymorphisms in VEGF gene are associated with an increased risk of osteosarcoma [J]. Int J Clin Exp Pathol, 2014, 7(11): 8143-9.

[33] Zhang Y, Hu X, Wang HK, Shen WW, Liao TQ, Chen P, et al. Single-nucleotide polymorphisms of the PRKCG gene and osteosarcoma susceptibility [J]. Tumour Biol, 2014, 35(12): 12671-7.

[34] Jiang C, Chen H, Shao L, Dong Y. GRM4 gene polymorphism is associated with susceptibility and prognosis of osteosarcoma in a China Han population [J]. Med Oncol, 2014, 31(7): 50.

[35] Wang Z, Wen P, Luo X, Fang X, Wang Q, Ma F, et al. Association of the vascular endothelial growth factor (VEGF) gene single-nucleotide polymorphisms with osteosarcoma susceptibility in a China population [J]. Tumour Biol, 2014, 35(4): 3605-10.

[36] Zhi LQ, Ma W, Zhang H, Zeng SX, Chen B. Association of RECQL5 gene polymorphisms and osteosarcoma in a China Han population [J]. Tumour Biol, 2014, 35(4): 3255-9.

[37] Xu S, Yang S, Sun G, Huang W, Zhang Y. Transforming growth factor-beta polymorphisms and serum level in the development of osteosarcoma [J]. DNA Cell Biol, 2014, 33(11): 802-6.

[38] Liu Y, He Z, Feng D, Shi G, Gao R, Wu X, et al. Cytotoxic T-lymphocyte antigen-4 polymorphisms and susceptibility to osteosarcoma [J]. DNA Cell Biol, 2011, 30(12): 1051-5.

[39] Wang W, Wang J, Song H, Liu J, Song B, Cao X. Cytotoxic T-lymphocyte antigen-4 +49G/A polymorphism is associated with increased risk of osteosarcoma [J]. Genet Test Mol Biomarkers, 2011, 15(7-8): 503-6.

[40] Biason P, Hattinger CM, Innocenti F, Talamini R, Alberghini M, Scotlandi K, et al. Nucleotide excision repair gene variants and association with survival in osteosarcoma patients treated with neoadjuvant chemotherapy [J]. Pharmacogenomics J, 2012, 12(6): 476-83.

[41] Toffoli G, Biason P, Russo A, De Mattia E, Cecchin E, Hattinger CM, et al. Effect of TP53 Arg72Pro and MDM2 SNP309 polymorphisms on the risk of high-grade osteosarcoma development and survival [J]. Clin Cancer Res, 2009, 15(10): 3550-6.

[42] Oliveira ID, Petrilli AS, Tavela MH, Zago MA, De Toledo SR. TNF-alpha, TNF-beta, IL6, IL10, PECAM-1 and the MPO inflammatory gene polymorphisms in osteosarcoma [J]. J Pediatr Hematol Oncol, 2007, 29(5): 293-7.

[43] Savage SA, Burdett L, Troisi R, Douglass C, Hoover RN, Chanock SJ. Germ-line genetic variation of TP53 in osteosarcoma [J]. Pediatr Blood Cancer, 2007, 49(1): 28-33.

**Supplementary Table S2. Characteristics of the included articles.**

| Ref Number^a^ | PMID | First author | Year | Ethnicity | Country/region | Study design | Dataset^b^ | Variant | Allelic | Gene name | Case | Control | Sample size |
| --- | --- | --- | --- | --- | --- | --- | --- | --- | --- | --- | --- | --- | --- |
| 1 | 30464596 | Wu Y | 2018 | Asian | China | CCS | 3 | rs1799782 | G>A | XRCC1 | 146 | 248 | 394 |
|  |  |  |  |  |  |  |  | rs25489 | C>T | XRCC1 | 146 | 248 | 394 |
|  |  |  |  |  |  |  |  | rs25487 | T>C | XRCC1 | 146 | 253 | 399 |
| 2 | 29595404 | Zhang J I^*^ | 2018 | Asian | China | CCS | 2 | rs3787547 | G>A | BCAS1 | 500 | 500 | 1000 |
|  |  |  |  |  |  |  |  | rs1295925 | T>C | VMP1 | 500 | 500 | 1000 |
| 2 | 29595404 | Zhang J II^＃^ | 2018 | Asian | China | CCS | 2 | rs3787547 | G>A | BCAS1 | 799 | 800 | 1599 |
|  |  |  |  |  |  |  |  | rs1295925 | T>C | VMP1 | 800 | 800 | 1600 |
| 3 | 28474168 | Xu Q | 2017 | Asian | China | CCS | 5 | rs11615 | A>G | ERCC1 | 381 | 765 | 1146 |
|  |  |  |  |  |  |  |  | rs13181 | T>G | ERCC2 | 381 | 765 | 1146 |
|  |  |  |  |  |  |  |  | rs1799793 | C>A | ERCC2 | 381 | 765 | 1146 |
|  |  |  |  |  |  |  |  | rs4150441 | T>C | ERCC3 | 381 | 765 | 1146 |
|  |  |  |  |  |  |  |  | rs4150506 | G>A | ERCC3 | 381 | 765 | 1146 |
| 4 | 28052683 | Bilbao-Aldaiturriaga N | 2017 | Caucasian | Spanish | CCS | 1 | rs231775 | A>G | CTLA4 | 66 | 125 | 191 |
| 5 | NA | Qiao G | 2017 | Asian | China | CCS | 2 | rs231775 | A>G | CTLA4 | 122 | 131 | 253 |
|  |  |  |  |  |  |  |  | rs5742909 | C>T | CTLA4 | 122 | 131 | 253 |
| 6 | NA | Niu J | 2017 | Asian | China | CCS | 1 | rs1800795 | C>G | IL6 | 116 | 116 | 232 |
| 7 | 28839218 | Xiao X I^*^ | 2017 | Asian | China | CCS | 5 | rs1130409 | T>G | APE1 | 172 | 256 | 428 |
|  |  |  |  |  |  |  |  | rs1760944 | T>G | APE1 | 172 | 256 | 428 |
|  |  |  |  |  |  |  |  | rs2275008 | T>C | APE1 | 172 | 256 | 428 |
|  |  |  |  |  |  |  |  | rs17111750 | C>T | APE1 | 172 | 256 | 428 |
|  |  |  |  |  |  |  |  | rs1760941 | A>C | APE1 | 172 | 256 | 428 |
| 7 | 28839218 | Xiao X II^＃^ | 2017 | Asian | China | CCS | 5 | rs1130409 | T>G | APE1 | 206 | 360 | 566 |
|  |  |  |  |  |  |  |  | rs1760944 | T>G | APE1 | 206 | 360 | 566 |
|  |  |  |  |  |  |  |  | rs2275008 | T>C | APE1 | 206 | 360 | 566 |
|  |  |  |  |  |  |  |  | rs17111750 | C>T | APE1 | 206 | 306 | 512 |
|  |  |  |  |  |  |  |  | rs1760941 | A>C | APE1 | 206 | 360 | 566 |
| 8 | 27994653 | Zhi L I^*^ | 2016 | Asian | China | CCS | 7 | rs7023329 | A>G | MTAP | 284 | 981 | 1265 |
|  |  |  |  |  |  |  |  | rs7027989 | A>G | MTAP | 284 | 981 | 1265 |
|  |  |  |  |  |  |  |  | rs2165408 | A>G | MTAP | 284 | 981 | 1265 |
|  |  |  |  |  |  |  |  | rs7871477 | T>G | MTAP | 284 | 981 | 1265 |
|  |  |  |  |  |  |  |  | rs7867176 | A>G | MTAP | 284 | 981 | 1265 |
|  |  |  |  |  |  |  |  | rs1544195 | G>A | MTAP | 284 | 981 | 1265 |
|  |  |  |  |  |  |  |  | rs7874112 | A>G | MTAP | 284 | 981 | 1265 |
| 8 | 27994653 | Zhi L II^＃^ | 2016 | Asian | China | CCS | 7 | rs7023329 | A>G | MTAP | 108 | 597 | 705 |
|  |  |  |  |  |  |  |  | rs7027989 | A>G | MTAP | 108 | 597 | 705 |
|  |  |  |  |  |  |  |  | rs2165408 | A>G | MTAP | 108 | 597 | 705 |
|  |  |  |  |  |  |  |  | rs7871477 | T>G | MTAP | 108 | 597 | 705 |
|  |  |  |  |  |  |  |  | rs7867176 | A>G | MTAP | 108 | 597 | 705 |
|  |  |  |  |  |  |  |  | rs1544195 | G>A | MTAP | 108 | 597 | 705 |
|  |  |  |  |  |  |  |  | rs7874112 | A>G | MTAP | 108 | 597 | 705 |
| 9 | 27051024 | Ma X | 2016 | Asian | China | CCS | 4 | rs13181 | T>G | ERCC2 | 141 | 282 | 423 |
|  |  |  |  |  |  |  |  | rs1799793 | C>A | ERCC2 | 141 | 282 | 423 |
|  |  |  |  |  |  |  |  | rs4150441 | T>C | ERCC3 | 141 | 282 | 423 |
|  |  |  |  |  |  |  |  | rs4150506 | G>A | ERCC3 | 141 | 283 | 424 |
| 10 | 26967389 | Zhou Q I^*^ | 2016 | Asian | China | CCS | 2 | rs874945 | C>T | HOTAIR | 500 | 500 | 1000 |
|  |  |  |  |  |  |  |  | rs7958904 | C>G | HOTAIR | 500 | 500 | 1000 |
| 10 | 26967389 | Zhou Q II^＃^ | 2016 | Asian | China | CCS | 2 | rs874945 | C>T | HOTAIR | 400 | 400 | 800 |
|  |  |  |  |  |  |  |  | rs7958904 | C>G | HOTAIR | 400 | 400 | 800 |
| 11 | 26810186 | Qi Y | 2016 | Asian | China | CCS | 1 | rs1800795 | C>G | IL6 | 216 | 216 | 432 |
| 12 | 26615418 | Chen Y | 2016 | Asian | China | CCS | 1 | rs4073 | A>T | IL8 | 190 | 190 | 380 |
| 13 | 26503210 | Cui Y | 2016 | Asian | China | CCS | 1 | rs1800896 | T>C | IL10 | 260 | 260 | 520 |
| 14 | 26276359 | Wang K | 2016 | Asian | China | CCS | 1 | rs1906953 | C>T | GRM4 | 126 | 168 | 294 |
| 15 | NA | Xu T | 2016 | Asian | China | CCS | 1 | rs4073 | A>T | IL8 | 109 | 109 | 218 |
| 16 | NA | Wang Z | 2016 | Asian | China | CCS | 4 | rs1799782 | G>A | XRCC1 | 172 | 275 | 447 |
|  |  |  |  |  |  |  |  | rs25489 | C>T | XRCC1 | 172 | 278 | 450 |
|  |  |  |  |  |  |  |  | rs25487 | T>C | XRCC1 | 172 | 275 | 447 |
|  |  |  |  |  |  |  |  | rs13181 | T>G | ERCC2 | 172 | 276 | 448 |
| 17 | NA | Mei J | 2016 | Asian | China | CCS | 1 | rs5742909 | C>T | CTLA4 | 97 | 120 | 217 |
| 18 | NA | Cao L | 2016 | Asian | China | CCS | 2 | rs2010963 | C>G | VEGF | 322 | 343 | 665 |
|  |  |  |  |  |  |  |  | rs3025039 | C>T | VEGF | 322 | 343 | 665 |
| 19 | 26662447 | Guo J | 2015 | Asian | China | CCS | 1 | rs861539 | G>A | XRCC3 Thr241Met | 136 | 136 | 272 |
| 20 | 26622695 | Liu J | 2015 | Asian | China | CCS | 5 | rs699947 | A>C | VEGF | 186 | 186 | 372 |
|  |  |  |  |  |  |  |  | rs1570360 | A>G | VEGF | 187 | 186 | 373 |
|  |  |  |  |  |  |  |  | rs10434 | A>G | VEGF | 186 | 186 | 372 |
|  |  |  |  |  |  |  |  | rs3025039 | C>T | VEGF | 187 | 186 | 373 |
|  |  |  |  |  |  |  |  | rs2010963 | C>G | VEGF | 186 | 186 | 372 |
| 21 | 26617908 | Yang L | 2015 | Asian | China | CCS | 1 | rs861539 | G>A | XRCC3 Thr241Met | 151 | 304 | 455 |
| 22 | 26600515 | Hu G | 2015 | Asian | China | CCS | 3 | rs2010963 | C>G | VEGF | 130 | 129 | 259 |
|  |  |  |  |  |  |  |  | rs3025039 | C>T | VEGF | 130 | 130 | 260 |
|  |  |  |  |  |  |  |  | rs10434 | A>G | VEGF | 130 | 130 | 260 |
| 23 | 26400284 | Zhang H | 2015 | Asian | China | CCS | 3 | rs699947 | A>C | VEGF | 182 | 182 | 364 |
|  |  |  |  |  |  |  |  | rs3025039 | C>T | VEGF | 182 | 182 | 364 |
|  |  |  |  |  |  |  |  | rs833061 | C>T | VEGF | 182 | 182 | 364 |
| 24 | 26345754 | Zhang G | 2015 | Asian | China | CCS | 3 | rs2010963 | C>G | VEGF | 180 | 361 | 541 |
|  |  |  |  |  |  |  |  | rs3025039 | C>T | VEGF | 180 | 360 | 540 |
|  |  |  |  |  |  |  |  | rs10434 | A>G | VEGF | 180 | 360 | 540 |
| 25 | 26101492 | Zhao L | 2015 | Asian | China | CCS | 6 | rs699947 | A>C | VEGF | 176 | 176 | 352 |
|  |  |  |  |  |  |  |  | rs1570360 | A>G | VEGF | 177 | 176 | 353 |
|  |  |  |  |  |  |  |  | rs10434 | A>G | VEGF | 176 | 176 | 352 |
|  |  |  |  |  |  |  |  | rs3025039 | C>T | VEGF | 176 | 176 | 352 |
|  |  |  |  |  |  |  |  | rs2010963 | C>G | VEGF | 176 | 176 | 352 |
|  |  |  |  |  |  |  |  | rs833061 | C>T | VEGF | 176 | 176 | 352 |
| 26 | 26097610 | Zhao Z | 2015 | Asian | China | CCS | 1 | rs1800629 | G>A | TNF-α | 80 | 99 | 179 |
| 27 | 26045840 | Ru J | 2015 | Asian | China | CCS | 2 | rs1042522 | G>C | TP53 | 209 | 420 | 629 |
|  |  |  |  |  |  |  |  | rs9895829 | A>G | TP53 | 209 | 420 | 629 |
| 28 | 25867335 | Dong YZ | 2015 | Asian | China | CCS | 1 | rs820196 | T>C | RECQL5 | 185 | 201 | 386 |
| 29 | 25789018 | Gómez-Díaz B | 2015 | Mixed | Mexican‑mestizo | CCS | 3 | rs11615 | A>G | ERCC1 | 28 | 97 | 125 |
|  |  |  |  |  |  |  |  | rs13181 | T>G | ERCC2 | 28 | 97 | 125 |
|  |  |  |  |  |  |  |  | rs1799793 | C>A | ERCC2 | 28 | 97 | 125 |
| 30 | 25663494 | Lu H | 2015 | Asian | China | CCS | 3 | rs454006 | T>C | PRKCG | 388 | 388 | 776 |
|  |  |  |  |  |  |  |  | rs8103851 | C>G | PRKCG | 388 | 388 | 776 |
|  |  |  |  |  |  |  |  | rs2242245 | T>C | PRKCG | 388 | 388 | 776 |
| 31 | 25663491 | Wu Y | 2015 | Asian | China | CCS | 2 | rs1800470 | G>A | TGF-β1 | 124 | 136 | 260 |
|  |  |  |  |  |  |  |  | rs1800469 | A>G | TGF-β1 | 124 | 136 | 260 |
| 32 | 25550863 | Tie Z | 2014 | Asian | China | CCS | 5 | rs699947 | A>C | VEGF | 165 | 330 | 495 |
|  |  |  |  |  |  |  |  | rs1570360 | A>G | VEGF | 165 | 330 | 495 |
|  |  |  |  |  |  |  |  | rs10434 | A>G | VEGF | 164 | 330 | 494 |
|  |  |  |  |  |  |  |  | rs3025039 | C>T | VEGF | 165 | 330 | 495 |
|  |  |  |  |  |  |  |  | rs2010963 | C>G | VEGF | 165 | 330 | 495 |
| 33 | 25252845 | Zhang Y | 2014 | Asian | China | CCS | 3 | rs454006 | T>C | PRKCG | 610 | 610 | 1220 |
|  |  |  |  |  |  |  |  | rs2242245 | T>C | PRKCG | 610 | 610 | 1220 |
|  |  |  |  |  |  |  |  | rs8103851 | C>G | PRKCG | 610 | 610 | 1220 |
| 34 | 24984297 | Jiang C | 2014 | Asian | China | CCS | 1 | rs1906953 | C>T | GRM4 | 168 | 216 | 384 |
| 35 | 24310504 | Wang Z | 2014 | Asian | China | CCS | 3 | rs2010963 | C>G | VEGF | 330 | 342 | 672 |
|  |  |  |  |  |  |  |  | rs3025039 | C>T | VEGF | 330 | 342 | 672 |
|  |  |  |  |  |  |  |  | rs10434 | A>G | VEGF | 330 | 342 | 672 |
| 36 | 24287950 | Zhi L | 2014 | Asian | China | CCS | 1 | rs820196 | T>C | RECQL5 | 212 | 240 | 452 |
| 37 | 25098449 | Xu S | 2014 | Asian | China | CCS | 2 | rs1800469 | A>G | TGF-β1 | 202 | 216 | 418 |
|  |  |  |  |  |  |  |  | rs1800470 | G>A | TGF-β1 | 202 | 216 | 418 |
| 38 | 21612409 | Liu Y | 2011 | Asian | China | CCS | 2 | rs5742909 | C>T | CTLA4 | 267 | 282 | 549 |
|  |  |  |  |  |  |  |  | rs231775 | A>G | CTLA4 | 267 | 282 | 549 |
| 39 | 21453059 | Wang W | 2011 | Asian | China | CCS | 1 | rs231775 | A>G | CTLA4 | 205 | 216 | 421 |
| 40 | 21826087 | Biason P | 2011 | Caucasian | Italy | CCS | 3 | rs13181 | T>G | ERCC2 | 130 | 250 | 380 |
|  |  |  |  |  |  |  |  | rs1799793 | C>A | ERCC2 | 130 | 250 | 380 |
|  |  |  |  |  |  |  |  | rs11615 | A>G | ERCC1 | 126 | 250 | 376 |
| 41 | 19451596 | Toffoli G | 2009 | Caucasian | Italy | CCS | 1 | rs1042522 | G>C | TP53 | 201 | 250 | 451 |
| 42 | 17483704 | Oliveira I | 2007 | Mixed | Brazil | CCS | 3 | rs1800629 | G>A | TNF-α | 80 | 160 | 240 |
|  |  |  |  |  |  |  |  | rs1800795 | C>G | IL6 | 64 | 160 | 224 |
|  |  |  |  |  |  |  |  | rs1800896 | T>C | IL10 | 78 | 157 | 235 |
| 43 | 17096406 | Savage S | 2007 | Caucasian | USA | CCS | 1 | rs1042522 | G>C | TP53 | 98 | 67 | 165 |
|  |  |  |  |  |  |  |  | rs9895829 | A>G | TP53 | 101 | 71 | 172 |

Note: I^*^, stage I; II^＃^, stage II; CCS, case-control study; A, adenine; C, cytosine; G, guanine; T, thymine; NA, not applicable.

^a^References for the 43 included articles are presented in the **Supplementary Table S1**.

^b^Datasets represent the number of datasets in the original publications.

**Supplementary Table 3: Genetic associations with OS risk for all 46 variants in 21 genes analyzed in main meta-analysis under the different genetic models.**

| **Gene** | **Variant** | **Allelic^a^** | **Ethnicity** | **Number Evaluation** | | | **MAF** | **Effect model** | **Risk of Meta-Analysis** | | | | **Venice Criteria^b^** | **Venice Criteria Grade** | **Amount of Evidence** | | **Replication** | **Protection from Bias** | | **First Study** | **Reason for bias Exemption** | **Deviation from HWE** | **P_begg_** | **P_egger_** | **Low OR** | **FPRP values^c^** | **Credibility of Evidence** |
| --- | --- | --- | --- | --- | --- | --- | --- | --- | --- | --- | --- | --- | --- | --- | --- | --- | --- | --- | --- | --- | --- | --- | --- | --- | --- | --- | --- |
|  |  |  |  | **Studies** | **Cases/controls** | **Genetic Models** |  |  | **OR (95%CI)** | **I^2^** | **P_Q_** | **P_value_** |  |  | **Nminor** | **Grade** |  | **Grade** | **Reasons for Bias** |  |  |  |  |  |  |  |  |
| **APEX1** | **rs1130409** | **T>G** | **Asian** | **2** | **378/616** | **Allelic** | **0.429** | **fixed** | **0.926 (0.771-1.113)** | **0** | **0.901** | **0.415** |  |  |  |  |  |  |  |  |  |  |  |  |  |  |  |
|  |  |  |  |  |  | **Dominant** |  | **fixed** | **0.933 (0.713-1.221)** | **0** | **0.9** | **0.612** |  |  |  |  |  |  |  |  |  |  |  |  |  |  |  |
|  |  |  |  |  |  | **Recessive** |  | **fixed** | **0.868 (0.622-1.209)** | **0** | **0.949** | **0.401** |  |  |  |  |  |  |  |  |  |  |  |  |  |  |  |
|  | **rs17111750** | **C>T** | **Asian** | **2** | **378/616** | **Allelic** | **0.235** | **fixed** | **1.055(0.851-1.309)** | **0** | **0.756** | **0.624** |  |  |  |  |  |  |  |  |  |  |  |  |  |  |  |
|  |  |  |  |  |  | **Dominant** |  | **fixed** | **0.978(0.749-1.277)** | **0** | **0.907** | **0.871** |  |  |  |  |  |  |  |  |  |  |  |  |  |  |  |
|  |  |  |  |  |  | **Recessive** |  | **fixed** | **1.379(0.866-2.195)** | **0** | **0.656** | **0.176** |  |  |  |  |  |  |  |  |  |  |  |  |  |  |  |
|  | **rs1760941** | **A>C** | **Asian** | **2** | **378/616** | **Allelic** | **0.715** | **fixed** | **1.145(0.934-1.405)** | **0** | **0.639** | **0.193** |  |  |  |  |  |  |  |  |  |  |  |  |  |  |  |
|  |  |  |  |  |  | **Dominant** |  | **fixed** | **1.231(0.783-1.935)** | **0** | **0.548** | **0.368** |  |  |  |  |  |  |  |  |  |  |  |  |  |  |  |
|  |  |  |  |  |  | **Recessive** |  | **fixed** | **1.160(0.896-1.501)** | **0** | **0.794** | **0.26** |  |  |  |  |  |  |  |  |  |  |  |  |  |  |  |
|  | **rs1760944** | **T>G** | **Asian** | **2** | **378/616** | **Allelic** | **0.442** | **fixed** | **0.692(0.574-0.834)** | **0** | **0.701** | **<0.001** | **BAA** | **Moderate** | **812** | **B** | **A** | **A** | **No** | **NA** | **No** | **NA** | **1** | **NA** | **No** | **0.003** | **Strong** |
|  |  |  |  |  |  | **Dominant** |  | **fixed** | **0.610(0.468-0.796)** | **0** | **0.748** | **<0.001** | **BAA** | **Moderate** | **637** | **B** | **A** | **A** | **No** | **NA** | **No** | **NA** | **1** | **NA** | **No** | **0.020** | **Strong** |
|  |  |  |  |  |  | **Recessive** |  | **fixed** | **0.642(0.451-0.914)** | **0** | **0.867** | **0.014** | **BAA** | **Moderate** | **175** | **B** | **A** | **A** | **No** | **NA** | **No** | **NA** | **1** | **NA** | **No** | **0.388** | **Weak** |
|  | **rs2275008** | **T>C** | **Asian** | **2** | **378/616** | **Allelic** | **0.128** | **fixed** | **1.160(0.892-1.508)** | **0** | **0.577** | **0.268** |  |  |  |  |  |  |  |  |  |  |  |  |  |  |  |
|  |  |  |  |  |  | **Dominant** |  | **fixed** | **1.226(0.913-1.645)** | **0** | **0.595** | **0.176** |  |  |  |  |  |  |  |  |  |  |  |  |  |  |  |
|  |  |  |  |  |  | **Recessive** |  | **fixed** | **0.865(0.363-2.064)** | **0** | **0.779** | **0.744** |  |  |  |  |  |  |  |  |  |  |  |  |  |  |  |
| **BCAS1** | **rs3787547** | **G>A** | **Asian** | **2** | **1300/1300** | **Allelic** | **0.300** | **fixed** | **1.222（1.088-1.373）** | **0** | **0.703** | **0.001** | **AAA** | **Strong** | **1671** | **A** | **A** | **A** | **No** | **NA** | **No** | **NA** | **1** | **NA** | **No** | **0.014** | **Strong** |
|  |  |  |  |  |  | **Dominant** |  | **fixed** | **1.295（1.110-1.511）** | **0** | **0.694** | **0.001** | **AAA** | **Strong** | **1375** | **A** | **A** | **A** | **No** | **NA** | **No** | **NA** | **1** | **NA** | **No** | **0.020** | **Strong** |
|  |  |  |  |  |  | **Recessive** |  | **fixed** | **1.259（0.987-1.606）** | **0** | **0.867** | **0.063** |  |  |  |  |  |  |  |  |  |  |  |  |  |  |  |
| **CTLA4** | **rs231775** | **A>G** | **Overall** | **4** | **660/754** | **Allelic** | **0.623** | **fixed** | **0.725 (0.620-0.846)** | **0** | **0.96** | **<0.001** | **AAA** | **Strong** | **1689** | **A** | **A** | **A** | **No** | **No** | **No** | **No** | **0.734** | **0.531** | **No** | **0.001** | **Strong** |
|  |  |  |  |  |  | **Dominant** |  | **fixed** | **0.491(0.360-0.668)** | **0** | **0.981** | **<0.001** | **AAA** | **Strong** | **1198** | **A** | **A** | **A** | **No** | **No** | **No** | **No** | **1** | **0.663** | **No** | **0.004** | **Strong** |
|  |  |  |  |  |  | **Recessive** |  | **fixed** | **0.748（0.596-0.938）** | **29.9%** | **0.233** | **0.012** | **BBC** | **Weak** | **491** | **B** | **B** | **C** | **First study** | **Yes** | **No** | **No** | **1** | **0.281** | **No** | **0.212** | **Weak** |
|  |  |  | **Asian** | **3** | **594/629** | **Allelic** | **0.672** | **fixed** | **0.723（0.613-0.853）** | **0** | **0.862** | **<0.001** | **AAA** | **Strong** | **1556** | **A** | **A** | **A** | **No** | **No** | **No** | **No** | **0.296** | **0.283** | **No** | **0.003** | **Strong** |
|  |  |  |  |  |  | **Dominant** |  | **fixed** | **0.506（0.354-0.722）** | **0** | **0.97** | **<0.001** | **AAA** | **Strong** | **1076** | **A** | **A** | **A** | **No** | **No** | **No** | **No** | **1** | **0.567** | **No** | **0.049** | **Strong** |
|  |  |  |  |  |  | **Recessive** |  | **fixed** | **0.717（0.569-0.903）** | **0** | **0.711** | **0.005** | **BAA** | **Moderate** | **480** | **B** | **A** | **A** | **No** | **No** | **No** | **No** | **0.296** | **0.262** | **No** | **0.109** | **Moderate** |
|  |  |  | **Caucasian** | **1** | **66/125** | **Allelic** | **0.372** | **fixed** | **0.734(0.467-1.152)** | **Na** | **Na** | **0.179** |  |  |  |  |  |  |  |  |  |  |  |  |  |  |  |
|  |  |  |  |  |  | **Dominant** |  | **fixed** | **0.447(0.241-0.828)** | **Na** | **Na** | **0.01** |  |  |  |  |  |  |  |  |  |  |  |  |  |  |  |
|  |  |  |  |  |  | **Recessive** |  | **fixed** | **2.4（0.704-8.183）** | **Na** | **Na** | **0.162** |  |  |  |  |  |  |  |  |  |  |  |  |  |  |  |
|  | **rs5742909** | **C>T** | **Asian** | **3** | **486/533** | **Allelic** | **0.156** | **random** | **1.235 (0.837-1.822)** | **58.4%** | **0.091** | **0.287** |  |  |  |  |  |  |  |  |  |  |  |  |  |  |  |
|  |  |  |  |  |  | **Dominant** |  | **fixed** | **1.219 (0.934-1.592)** | **56.0%** | **0.103** | **0.145** |  |  |  |  |  |  |  |  |  |  |  |  |  |  |  |
|  |  |  |  |  |  | **Recessive** |  | **fixed** | **2.046(1.028-4.073)** | **0.0%** | **0.591** | **0.042** | **CAC** | **Weak** | **37** | **C** | **A** | **C** | **First study** | **Yes** | **No** | **None** | **0.296** | **0.188** | **No** | **0.807** | **Weak** |
| **ERCC1** | **rs11615** | **A>G** | **Overall** | **3** | **535/1112** | **Allelic** | **0.690** | **fixed** | **0.884(0.753-1.038)** | **55.5%** | **0.106** | **0.133** |  |  |  |  |  |  |  |  |  |  |  |  |  |  |  |
|  |  |  |  |  |  | **Dominant** |  | **fixed** | **0.893(0.648-1.231)** | **54.9%** | **0.109** | **0.491** |  |  |  |  |  |  |  |  |  |  |  |  |  |  |  |
|  |  |  |  |  |  | **Recessive** |  | **fixed** | **0.844（0.680-1.047）** | **0** | **0.392** | **0.123** |  |  |  |  |  |  |  |  |  |  |  |  |  |  |  |
|  |  |  | **Asian** | **1** | **381/765** | **Allelic** | **0.763** | **fixed** | **0.791(0.650-0.964)** | **Na** | **Na** | **0.02** |  |  |  |  |  |  |  |  |  |  |  |  |  |  |  |
|  |  |  |  |  |  | **Dominant** |  | **fixed** | **0.644(0.403-1.029)** | **Na** | **Na** | **0.066** |  |  |  |  |  |  |  |  |  |  |  |  |  |  |  |
|  |  |  |  |  |  | **Recessive** |  | **fixed** | **0.782（0.611-1.001）** | **Na** | **Na** | **0.051** |  |  |  |  |  |  |  |  |  |  |  |  |  |  |  |
|  |  |  | **Caucasian** | **1** | **126/250** | **Allelic** | **0.434** | **fixed** | **1.167(0.861-1.581)** | **Na** | **Na** | **0.32** |  |  |  |  |  |  |  |  |  |  |  |  |  |  |  |
|  |  |  |  |  |  | **Dominant** |  | **fixed** | **1.261(0.793-2.006)** | **Na** | **Na** | **0.326** |  |  |  |  |  |  |  |  |  |  |  |  |  |  |  |
|  |  |  |  |  |  | **Recessive** |  | **fixed** | **1.162（0.698-1.934）** | **Na** | **Na** | **0.565** |  |  |  |  |  |  |  |  |  |  |  |  |  |  |  |
|  |  |  | **Mixed** | **1** | **28/97** | **Allelic** | **0.773** | **fixed** | **0.802(0.406-1.583)** | **Na** | **Na** | **0.524** |  |  |  |  |  |  |  |  |  |  |  |  |  |  |  |
|  |  |  |  |  |  | **Dominant** |  | **fixed** | **0.549(0.128-2.354)** | **Na** | **Na** | **0.420** |  |  |  |  |  |  |  |  |  |  |  |  |  |  |  |
|  |  |  |  |  |  | **Recessive** |  | **fixed** | **0.859（0.366-2.014）** | **Na** | **Na** | **0.726** |  |  |  |  |  |  |  |  |  |  |  |  |  |  |  |
| **ERCC2** | **rs13181** | **T>G** | **Overall** | **5** | **852/1669** | **Allelic** | **0.483** | **random** | **0.978(0.787-1.215)** | **53.3%** | **0.073** | **0.839** |  |  |  |  |  |  |  |  |  |  |  |  |  |  |  |
|  |  |  |  |  |  | **Dominant** |  | **fixed** | **0.992(0.808-1.218)** | **21.8%** | **0.275** | **0.939** |  |  |  |  |  |  |  |  |  |  |  |  |  |  |  |
|  |  |  |  |  |  | **Recessive** |  | **fixed** | **0.865(0.703-1.066)** | **20.8%** | **0.282** | **0.174** |  |  |  |  |  |  |  |  |  |  |  |  |  |  |  |
|  |  |  | **Asian** | **3** | **694/1322** | **Allelic** | **0.521** | **random** | **1.043(0.755-1.439)** | **74.5%** | **0.020** | **0.800** |  |  |  |  |  |  |  |  |  |  |  |  |  |  |  |
|  |  |  |  |  |  | **Dominant** |  | **fixed** | **1.025(0.806-1.304)** | **53.3%** | **0.118** | **0.840** |  |  |  |  |  |  |  |  |  |  |  |  |  |  |  |
|  |  |  |  |  |  | **Recessive** |  | **fixed** | **0.881(0.706-1.101** | **58.7%** | **0.089** | **0.265** |  |  |  |  |  |  |  |  |  |  |  |  |  |  |  |
|  |  |  | **Caucasian** | **1** | **126/250** | **Allelic** | **0.396** | **random** | **0.923(0.678-1.256)** | **Na** | **Na** | **0.609** |  |  |  |  |  |  |  |  |  |  |  |  |  |  |  |
|  |  |  |  |  |  | **Dominant** |  | **fixed** | **0.979(0.632-1.517)** | **Na** | **Na** | **0.925** |  |  |  |  |  |  |  |  |  |  |  |  |  |  |  |
|  |  |  |  |  |  | **Recessive** |  | **fixed** | **0.767(0.417-1.411)** | **Na** | **Na** | **0.394** |  |  |  |  |  |  |  |  |  |  |  |  |  |  |  |
|  |  |  | **Mixed** | **1** | **28/97** | **Allelic** | **0.180** | **random** | **0.649(0.271-1.553)** | **Na** | **Na** | **0.331** |  |  |  |  |  |  |  |  |  |  |  |  |  |  |  |
|  |  |  |  |  |  | **Dominant** |  | **fixed** | **0.646(0.249-1.677)** | **Na** | **Na** | **0.370** |  |  |  |  |  |  |  |  |  |  |  |  |  |  |  |
|  |  |  |  |  |  | **Recessive** |  | **fixed** | **0.670(0.031-14.366)** | **Na** | **Na** | **0.798** |  |  |  |  |  |  |  |  |  |  |  |  |  |  |  |
| **ERCC2** | **rs1799793** | **C>A** | **Overall** | **4** | **680/1394** | **Allelic** | **0.331** | **random** | **0.938(0.533-1.652)** | **91.6%** | **0** | **0.826** |  |  |  |  |  |  |  |  |  |  |  |  |  |  |  |
|  |  |  |  |  |  | **Dominant** |  | **random** | **0.917(0.452-1.860)** | **88.3%** | **0** | **0.810** |  |  |  |  |  |  |  |  |  |  |  |  |  |  |  |
|  |  |  |  |  |  | **Recessive** |  | **random** | **1.046(0.504-2.171)** | **84.2%** | **0** | **0.903** |  |  |  |  |  |  |  |  |  |  |  |  |  |  |  |
|  |  |  | **Asian** | **2** | **522/1047** | **Allelic** | **0.333** | **random** | **1.240(0.597-2.575)** | **93.9%** | **0** | **0.564** |  |  |  |  |  |  |  |  |  |  |  |  |  |  |  |
|  |  |  |  |  |  | **Dominant** |  | **random** | **1.265(0.560-2.855)** | **83.8%** | **0.013** | **0.572** |  |  |  |  |  |  |  |  |  |  |  |  |  |  |  |
|  |  |  |  |  |  | **Recessive** |  | **random** | **1.459(0.466-4.565)** | **93.0%** | **0** | **0.516** |  |  |  |  |  |  |  |  |  |  |  |  |  |  |  |
|  |  |  | **Caucasian** | **1** | **130/250** | **Allelic** | **0.352** | **random** | **0.652(0.468-0.909)** | **Na** | **Na** | **0.012** |  |  |  |  |  |  |  |  |  |  |  |  |  |  |  |
|  |  |  |  |  |  | **Dominant** |  | **random** | **0.559(0.364-0.858)** | **Na** | **Na** | **0.008** |  |  |  |  |  |  |  |  |  |  |  |  |  |  |  |
|  |  |  |  |  |  | **Recessive** |  | **random** | **0.728(0.384-1.379)** | **Na** | **Na** | **0.330** |  |  |  |  |  |  |  |  |  |  |  |  |  |  |  |
|  |  |  | **Mixed** | **1** | **28/97** | **Allelic** | **0.258** | **random** | **0.704(0.338-1.466)** | **Na** | **Na** | **0.348** |  |  |  |  |  |  |  |  |  |  |  |  |  |  |  |
|  |  |  |  |  |  | **Dominant** |  | **random** | **0.782(0.299-2.040)** | **Na** | **Na** | **0.615** |  |  |  |  |  |  |  |  |  |  |  |  |  |  |  |
|  |  |  |  |  |  | **Recessive** |  | **random** | **0.603(0.188-1.931)** | **Na** | **Na** | **0.394** |  |  |  |  |  |  |  |  |  |  |  |  |  |  |  |
| **ERCC3** | **rs4150441** | **T>C** | **Asian** | **2** | **522/1047** | **Allelic** | **0.767** | **random** | **0.686(0.451-1.043)** | **81.7%** | **0.019** | **0.078** |  |  |  |  |  |  |  |  |  |  |  |  |  |  |  |
|  |  |  |  |  |  | **Dominant** |  | **fixed** | **0.519(0.357-0.755)** | **60.1%** | **0.113** | **0.001** | **ACA** | **Weak** | **1447** | **A** | **C** | **A** | **No** | **NA** | **No** | **NA** | **1** | **NA** | **No** | **0.108** | **Moderate** |
|  |  |  |  |  |  | **Recessive** |  | **random** | **0.651(0.408-1.038)** | **73.8%** | **0.051** | **0.071** |  |  |  |  |  |  |  |  |  |  |  |  |  |  |  |
|  | **rs4150506** | **G>A** | **Asian** | **2** | **522/1047** | **Allelic** | **0.230** | **fixed** | **1.331(1.123-1.576)** | **0** | **0.581** | **0.001** | **BAA** | **Moderate** | **777** | **B** | **A** | **A** | **No** | **NA** | **No** | **NA** | **1** | **NA** | **No** | **0.019** | **Strong** |
|  |  |  |  |  |  | **Dominant** |  | **fixed** | **1.348(1.089-1.667)** | **0** | **0.775** | **0.006** | **BAA** | **Moderate** | **658** | **B** | **A** | **A** | **No** | **NA** | **No** | **NA** | **1** | **NA** | **No** | **0.117** | **Moderate** |
|  |  |  |  |  |  | **Recessive** |  | **fixed** | **1.622(1.110-2.370)** | **0** | **0.498** | **0.012** | **BAA** | **Moderate** | **119** | **B** | **A** | **A** | **No** | **NA** | **No** | **NA** | **1** | **NA** | **No** | **0.408** | **Weak** |
| **GRM4** | **rs1906953** | **C>T** | **Asian** | **2** | **294/384** | **Allelic** | **0.486** | **random** | **1.013(0.426-2.410)** | **93.6%** | **0** | **0.977** |  |  |  |  |  |  |  |  |  |  |  |  |  |  |  |
|  |  |  |  |  |  | **Dominant** |  | **random** | **1.039(0.350-3.090)** | **88.9%** | **0.003** | **0.944** |  |  |  |  |  |  |  |  |  |  |  |  |  |  |  |
|  |  |  |  |  |  | **Recessive** |  | **random** | **0.995(0.300-3.301)** | **91.0%** | **0.001** | **0.994** |  |  |  |  |  |  |  |  |  |  |  |  |  |  |  |
| **HOTAIR** | **rs7958904** | **C>G** | **Asian** | **2** | **900/900** | **Allelic** | **0.710** | **fixed** | **1.294(1.115-1.501)** | **0** | **0.736** | **0.001** | **AAA** | **Strong** | **2646** | **A** | **A** | **A** | **No** | **NA** | **No** | **NA** | **1** | **NA** | **No** | **0.013** | **Strong** |
|  |  |  |  |  |  | **Dominant** |  | **fixed** | **1.636(1.154-2.321)** | **0** | **0.961** | **0.006** | **AAA** | **Strong** | **1656** | **A** | **A** | **A** | **No** | **NA** | **No** | **NA** | **1** | **NA** | **No** | **0.260** | **Moderate** |
|  |  |  |  |  |  | **Recessive** |  | **fixed** | **1.298(1.078-1.564)** | **0** | **0.768** | **0.006** | **BAA** | **Moderate** | **990** | **B** | **A** | **A** | **No** | **NA** | **No** | **NA** | **1** | **NA** | **No** | **0.110** | **Moderate** |
| **HOTAIR** | **rs874945** | **C>T** | **Asian** | **2** | **900/900** | **Allelic** | **0.189** | **fixed** | **1.183(1.006-1.393)** | **17.4%** | **0.271** | **0.042** | **BAA** | **Moderate** | **731** | **B** | **A** | **A** | **No** | **NA** | **No** | **NA** | **1** | **NA** | **No** | **0.455** | **Weak** |
|  |  |  |  |  |  | **Dominant** |  | **fixed** | **1.166(0.959-1.417)** | **10.3%** | **0.291** | **0.124** |  |  |  |  |  |  |  |  |  |  |  |  |  |  |  |
|  |  |  |  |  |  | **Recessive** |  | **fixed** | **1.397(0.955-2.044)** | **0** | **0.605** | **0.085** |  |  |  |  |  |  |  |  |  |  |  |  |  |  |  |
| **IL10** | **rs1800896** | **T>C** | **Overall** | **2** | **340/420** | **Allelic** | **0.391** | **fixed** | **1.326（1.060-1.657）** | **0** | **0.557** | **0.013** | **BAA** | **Moderate** | **597** | **B** | **A** | **A** | **No** | **NA** | **No** | **NA** | **1** | **NA** | **No** | **0.224** | **Weak** |
|  |  |  |  |  |  | **Dominant** |  | **fixed** | **1.398（1.009-1.936）** | **33.9%** | **0.219** | **0.044** | **BBA** | **Moderate** | **433** | **B** | **B** | **A** | **No** | **NA** | **No** | **NA** | **1** | **NA** | **No** | **0.556** | **Weak** |
|  |  |  |  |  |  | **Recessive** |  | **fixed** | **1.402（0.970-2.027）** | **56.4%** | **0.130** | **0.072** |  |  |  |  |  |  |  |  |  |  |  |  |  |  |  |
|  |  |  | **Asian** | **1** | **260/260** | **Allelic** | **0.258** | **fixed** | **1.387（1.060-1.815）** | **Na** | **Na** | **0.017** |  |  |  |  |  |  |  |  |  |  |  |  |  |  |  |
|  |  |  |  |  |  | **Dominant** |  | **fixed** | **1.285（0.908-1.819）** | **Na** | **Na** | **0.157** |  |  |  |  |  |  |  |  |  |  |  |  |  |  |  |
|  |  |  |  |  |  | **Recessive** |  | **fixed** | **1.804（1.097-2.965）** | **Na** | **Na** | **0.020** |  |  |  |  |  |  |  |  |  |  |  |  |  |  |  |
|  |  |  | **Mixed** | **1** | **80/160** | **Allelic** | **0.611** | **fixed** | **1.200（0.804-1.791）** | **Na** | **Na** | **0.372** |  |  |  |  |  |  |  |  |  |  |  |  |  |  |  |
|  |  |  |  |  |  | **Dominant** |  | **fixed** | **2.506（0.914-6.868）** | **Na** | **Na** | **0.074** |  |  |  |  |  |  |  |  |  |  |  |  |  |  |  |
|  |  |  |  |  |  | **Recessive** |  | **fixed** | **1.010（0.576-1.772）** | **Na** | **Na** | **0.972** |  |  |  |  |  |  |  |  |  |  |  |  |  |  |  |
| **IL6** | **rs1800795** | **C>G** | **Overall** | **3** | **402/482** | **Allelic** | **0.596** | **random** | **0.715(0.445-1.149)** | **0** | **0.008** | **0.166** |  |  |  |  |  |  |  |  |  |  |  |  |  |  |  |
|  |  |  |  |  |  | **Dominant** |  | **fixed** | **0.832(0.588-1.176)** | **38.5%** | **0.197** | **0.297** |  |  |  |  |  |  |  |  |  |  |  |  |  |  |  |
|  |  |  |  |  |  | **Recessive** |  | **random** | **0.644(0.279-1.579)** | **85.0%** | **0.001** | **0.355** |  |  |  |  |  |  |  |  |  |  |  |  |  |  |  |
|  |  |  | **Asian** | **2** | **322/322** | **Allelic** | **0.750** | **random** | **0.563(0.445-0.712)** | **0** | **0.805** | **<0.001** | **BAA** | **Moderate** | **915** | **B** | **A** | **A** | **No** | **NA** | **No** | **NA** | **1** | **NA** | **No** | **0.000** | **Strong** |
|  |  |  |  |  |  | **Dominant** |  | **fixed** | **0.730(0.473-1.126)** | **56.4%** | **0.130** | **0.155** |  |  |  |  |  |  |  |  |  |  |  |  |  |  |  |
|  |  |  |  |  |  | **Recessive** |  | **random** | **0.420(0.268-0.659)** | **47.6%** | **0.167** | **<0.001** | **BBA** | **Moderate** | **348** | **B** | **B** | **A** | **No** | **NA** | **No** | **NA** | **1** | **NA** | **No** | **0.121** | **Moderate** |
|  |  |  | **Mixed** | **1** | **80/160** | **Allelic** | **0.275** | **random** | **1.242(0.445-1.149)** | **Na** | **Na** | **0.339** |  |  |  |  |  |  |  |  |  |  |  |  |  |  |  |
|  |  |  |  |  |  | **Dominant** |  | **fixed** | **1.051(0.589-1.877)** | **Na** | **Na** | **0.866** |  |  |  |  |  |  |  |  |  |  |  |  |  |  |  |
|  |  |  |  |  |  | **Recessive** |  | **random** | **2.455(0.947-6.360)** | **Na** | **Na** | **0.065** |  |  |  |  |  |  |  |  |  |  |  |  |  |  |  |
| **IL8** | **rs4073** | **A>T** | **Asian** | **2** | **299/299** | **Allelic** | **0.776** | **fixed** | **0.625(0.483-0.809)** | **0** | **0.793** | **<0.001** | **BAA** | **Moderate** | **873** | **B** | **A** | **A** | **No** | **NA** | **No** | **NA** | **1** | **NA** | **No** | **0.021** | **Strong** |
|  |  |  |  |  |  | **Dominant** |  | **fixed** | **0.598(0.366-0.975)** | **0** | **0.949** | **0.039** | **BAA** | **Moderate** | **521** | **B** | **A** | **A** | **No** | **NA** | **No** | **NA** | **1** | **NA** | **No** | **0.692** | **Weak** |
|  |  |  |  |  |  | **Recessive** |  | **fixed** | **0.590(0.424-0.819)** | **0** | **0.823** | **0.002** | **BAA** | **Moderate** | **352** | **B** | **A** | **A** | **No** | **NA** | **No** | **NA** | **1** | **NA** | **No** | **0.116** | **Moderate** |
| **MTAP** | **rs1544195** | **G>A** | **Asian** | **2** | **392/1578** | **Allelic** | **0.080** | **fixed** | **1.111（0.840-1.470）** | **0** | **0.892** | **0.460** |  |  |  |  |  |  |  |  |  |  |  |  |  |  |  |
|  |  |  |  |  |  | **Dominant** |  | **fixed** | **1.102（0.816-1.489）** | **0** | **0.900** | **0.525** |  |  |  |  |  |  |  |  |  |  |  |  |  |  |  |
|  |  |  |  |  |  | **Recessive** |  | **fixed** | **1.457（0.459-4.629）** | **0** | **0.958** | **0.523** |  |  |  |  |  |  |  |  |  |  |  |  |  |  |  |
|  | **rs2165408** | **A>G** | **Asian** | **2** | **392/1578** | **Allelic** | **0.747** | **fixed** | **0.924（0.774-1.104）** | **0** | **0.938** | **0.386** |  |  |  |  |  |  |  |  |  |  |  |  |  |  |  |
|  |  |  |  |  |  | **Dominant** |  | **fixed** | **0.847（0.555-1.294）** | **0** | **0.828** | **0.443** |  |  |  |  |  |  |  |  |  |  |  |  |  |  |  |
|  |  |  |  |  |  | **Recessive** |  | **fixed** | **0.925（0.740-1.156）** | **0** | **0.979** | **0.491** |  |  |  |  |  |  |  |  |  |  |  |  |  |  |  |
|  | **rs7023329** | **A>G** | **Asian** | **2** | **392/1578** | **Allelic** | **0.512** | **fixed** | **0.712（0.615-0.844）** | **0** | **0.540** | **<0.001** | **AAA** | **Strong** | **1954** | **A** | **A** | **A** | **No** | **NA** | **No** | **NA** | **1** | **NA** | **No** | **0.002** | **Strong** |
|  |  |  |  |  |  | **Dominant** |  | **fixed** | **0.650（0.510-0.828）** | **0** | **0.439** | **<0.001** | **AAA** | **Strong** | **1470** | **A** | **A** | **A** | **No** | **NA** | **No** | **NA** | **1** | **NA** | **No** | **0.022** | **Strong** |
|  |  |  |  |  |  | **Recessive** |  | **fixed** | **0.641（0.484-0.848）** | **0** | **0.855** | **0.002** | **BAA** | **Moderate** | **484** | **B** | **A** | **A** | **No** | **NA** | **No** | **NA** | **1** | **NA** | **No** | **0.082** | **Moderate** |
|  | **rs7027989** | **A>G** | **Asian** | **2** | **392/1578** | **Allelic** | **0.824** | **fixed** | **0.761（0.627-0.923）** | **0** | **0.905** | **0.006** | **AAA** | **Strong** | **3216** | **A** | **A** | **A** | **No** | **NA** | **No** | **NA** | **1** | **NA** | **No** | **0.104** | **Strong** |
|  |  |  |  |  |  | **Recessive** |  | **fixed** | **0.757（0.601-0.954）** | **0** | **0.760** | **0.018** | **AAA** | **Strong** | **1315** | **A** | **A** | **A** | **No** | **NA** | **No** | **NA** | **1** | **NA** | **No** | **0.288** | **Moderate** |
|  |  |  |  |  |  | **Dominant** |  | **fixed** | **0.557（0.328-0.945）** | **0** | **0.751** | **0.030** | **AAA** | **Strong** | **1901** | **A** | **A** | **A** | **No** | **NA** | **No** | **NA** | **1** | **NA** | **No** | **0.693** | **Moderate** |
|  | **rs7867176** | **A>G** | **Asian** | **2** | **392/1578** | **Allelic** | **0.323** | **fixed** | **0.982（0.830-1.162）** | **0** | **0.920** | **0.834** |  |  |  |  |  |  |  |  |  |  |  |  |  |  |  |
|  |  |  |  |  |  | **Dominant** |  | **fixed** | **0.946（0.757-1.182）** | **0** | **0.904** | **0.624** |  |  |  |  |  |  |  |  |  |  |  |  |  |  |  |
|  |  |  |  |  |  | **Recessive** |  | **fixed** | **1.065（0.745-1.522）** | **0** | **0.985** | **0.729** |  |  |  |  |  |  |  |  |  |  |  |  |  |  |  |
|  | **rs7871477** | **T>G** | **Asian** | **2** | **392/1578** | **Allelic** | **0.276** | **fixed** | **1.055（0.887-1.256）** | **0** | **0.960** | **0.545** |  |  |  |  |  |  |  |  |  |  |  |  |  |  |  |
|  |  |  |  |  |  | **Dominant** |  | **fixed** | **1.065（0.853-1.330）** | **0** | **0.882** | **0.580** |  |  |  |  |  |  |  |  |  |  |  |  |  |  |  |
|  |  |  |  |  |  | **Recessive** |  | **fixed** | **1.087（0.722-1.635）** | **0** | **0.877** | **0.690** |  |  |  |  |  |  |  |  |  |  |  |  |  |  |  |
|  | **rs7874112** | **A>G** | **Asian** | **2** | **392/1578** | **Allelic** | **0.257** | **fixed** | **1.091(0.915-1.302)** | **0** | **0.938** | **0.332** |  |  |  |  |  |  |  |  |  |  |  |  |  |  |  |
|  |  |  |  |  |  | **Dominant** |  | **fixed** | **1.118(0.895-1.397)** | **0** | **0.979** | **0.324** |  |  |  |  |  |  |  |  |  |  |  |  |  |  |  |
|  |  |  |  |  |  | **Recessive** |  | **fixed** | **1.104(0.714-1.706)** | **0** | **0.888** | **0.657** |  |  |  |  |  |  |  |  |  |  |  |  |  |  |  |
| **PRKCG** | **rs2242245** | **T>C** | **Asian** | **2** | **998/998** | **Allelic** | **0.161** | **fixed** | **1.083(0.917-1.279)** | **0** | **0.655** | **0.350** |  |  |  |  |  |  |  |  |  |  |  |  |  |  |  |
|  |  |  |  |  |  | **Dominant** |  | **fixed** | **1.044(0.861-1.267)** | **0** | **0.519** | **0.658** |  |  |  |  |  |  |  |  |  |  |  |  |  |  |  |
|  |  |  |  |  |  | **Recessive** |  | **fixed** | **1.413(0.896-2.229)** | **0** | **0.860** | **0.137** |  |  |  |  |  |  |  |  |  |  |  |  |  |  |  |
|  | **rs454006** | **T>C** | **Asian** | **2** | **998/998** | **Allelic** | **0.293** | **fixed** | **1.347(1.178-1.539)** | **0** | **0.826** | **<0.001** | **AAA** | **Strong** | **1299** | **A** | **A** | **A** | **No** | **NA** | **No** | **NA** | **1** | **NA** | **No** | **0.000** | **Strong** |
|  |  |  |  |  |  | **Dominant** |  | **fixed** | **1.204(1.010-1.437)** | **15.4%** | **0.277** | **0.039** | **AAA** | **Strong** | **1006** | **A** | **A** | **A** | **No** | **NA** | **No** | **NA** | **1** | **NA** | **No** | **0.432** | **Moderate** |
|  |  |  |  |  |  | **Recessive** |  | **fixed** | **1.989(1.536-2.575)** | **0** | **0.596** | **<0.001** | **BAA** | **Moderate** | **293** | **B** | **A** | **A** | **No** | **NA** | **No** | **NA** | **1** | **NA** | **No** | **0.000** | **Strong** |
|  | **rs8103851** | **C>G** | **Asian** | **2** | **998/998** | **Allelic** | **0.444** | **fixed** | **0.939(0.828-1.064)** | **47.4%** | **0.168** | **0.323** |  |  |  |  |  |  |  |  |  |  |  |  |  |  |  |
|  |  |  |  |  |  | **Dominant** |  | **fixed** | **0.943(0.784-1.136)** | **0** | **0.432** | **0.538** |  |  |  |  |  |  |  |  |  |  |  |  |  |  |  |
|  |  |  |  |  |  | **Recessive** |  | **fixed** | **0.897(0.723-1.113)** | **54.4%** | **0.139** | **0.322** |  |  |  |  |  |  |  |  |  |  |  |  |  |  |  |
| **RECQL5** | **rs820196** | **T>C** | **Asian** | **2** | **397/441** | **Allelic** | **0.340** | **fixed** | **1.445(1.186-1.762)** | **0** | **0.742** | **<0.001** | **BAA** | **Moderate** | **639** | **B** | **A** | **A** | **No** | **NA** | **No** | **NA** | **1** | **NA** | **No** | **0.008** | **Strong** |
|  |  |  |  |  |  | **Dominant** |  | **fixed** | **1.487(1.118-1.976)** | **0** | **0.844** | **0.006** | **BAA** | **Moderate** | **534** | **B** | **A** | **A** | **No** | **NA** | **No** | **NA** | **1** | **NA** | **No** | **0.184** | **Moderate** |
|  |  |  |  |  |  | **Recessive** |  | **fixed** | **2.153(1.409-3.289)** | **0** | **0.700** | **<0.001** | **BAA** | **Moderate** | **105** | **B** | **A** | **A** | **No** | **NA** | **No** | **NA** | **1** | **NA** | **No** | **0.135** | **Moderate** |
| **TGF-β1** | **rs1800469** | **A>G** | **Asian** | **2** | **326/352** | **Allelic** | **0.531** | **fixed** | **0.977(0.789-1.211)** | **0** | **0.586** | **0.832** |  |  |  |  |  |  |  |  |  |  |  |  |  |  |  |
|  |  |  |  |  |  | **Dominant** |  | **fixed** | **0.884(0.616-1.268)** | **0** | **0.811** | **0.502** |  |  |  |  |  |  |  |  |  |  |  |  |  |  |  |
|  |  |  |  |  |  | **Recessive** |  | **fixed** | **1.050(0.753-1.464)** | **32.3%** | **0.224** | **0.772** |  |  |  |  |  |  |  |  |  |  |  |  |  |  |  |
|  | **rs1800470** | **G>A** | **Asian** | **2** | **326/352** | **Allelic** | **0.533** | **random** | **1.019(0.442-2.352)** | **93.0%** | **0** | **0.964** |  |  |  |  |  |  |  |  |  |  |  |  |  |  |  |
|  |  |  |  |  |  | **Dominant** |  | **random** | **0.906(0.348-2.356)** | **85.5%** | **0.009** | **0.840** |  |  |  |  |  |  |  |  |  |  |  |  |  |  |  |
|  |  |  |  |  |  | **Recessive** |  | **random** | **1.065(0.337-3.370)** | **91.2%** | **0.001** | **0.914** |  |  |  |  |  |  |  |  |  |  |  |  |  |  |  |
| **TNF-α** | **rs1800629** | **G>A** | **Overall** | **2** | **160/259** | **Allelic** | **0.183** | **fixed** | **1.743(1.245-2.440)** | **0** | **0.582** | **0.001** | **BAA** | **Moderate** | **192** | **B** | **A** | **A** | **No** | **NA** | **No** | **NA** | **1** | **NA** | **No** | **0.107** | **Moderate** |
|  |  |  |  |  |  | **Dominant** |  | **fixed** | **1.640(1.065-2.524)** | **0** | **0.427** | **0.025** | **BAA** | **Moderate** | **159** | **B** | **A** | **A** | **No** | **NA** | **No** | **NA** | **1** | **NA** | **No** | **0.576** | **Weak** |
|  |  |  |  |  |  | **Recessive** |  | **fixed** | **3.306(1.541-7.093)** | **0** | **0.588** | **0.002** | **CAA** | **Weak** | **33** | **C** | **A** | **A** | **No** | **NA** | **No** | **NA** | **1** | **NA** | **No** | **0.657** | **Weak** |
|  |  | **G>A** | **Asian** | **1** | **80/99** | **Allelic** | **0.303** | **fixed** | **1.615(1.043-2.500)** | **Na** | **Na** | **0.032** |  |  |  |  |  |  |  |  |  |  |  |  |  |  |  |
|  |  |  |  |  |  | **Dominant** |  | **fixed** | **1.389(0.761-2.534)** | **Na** | **Na** | **0.284** |  |  |  |  |  |  |  |  |  |  |  |  |  |  |  |
|  |  |  |  |  |  | **Recessive** |  | **fixed** | **3.875(1.439-10.437)** | **Na** | **Na** | **0.007** |  |  |  |  |  |  |  |  |  |  |  |  |  |  |  |
|  |  | **G>A** | **Mixed** | **1** | **80/160** | **Allelic** | **0.109** | **fixed** | **1.957(1.156-3.312)** | **Na** | **Na** | **0.012** |  |  |  |  |  |  |  |  |  |  |  |  |  |  |  |
|  |  |  |  |  |  | **Dominant** |  | **fixed** | **1.970(1.062-3.652)** | **Na** | **Na** | **0.031** |  |  |  |  |  |  |  |  |  |  |  |  |  |  |  |
|  |  |  |  |  |  | **Recessive** |  | **fixed** | **2.514(0.743-8.503)** | **Na** | **Na** | **0.138** |  |  |  |  |  |  |  |  |  |  |  |  |  |  |  |
| **TP53** | **rs1042522** | **G>C** | **Overall** | **3** | **515/744** | **Allelic** | **0.499** | **fixed** | **0.738(0.618-0.881)** | **0.0%** | **0.754** | **0.001** | **AAA** | **Strong** | **1169** | **A** | **A** | **A** | **No** | **No** | **No** | **No** | **0.296** | **0.302** | **No** | **0.017** | **Strong** |
|  |  |  |  |  |  | **Dominant** |  | **fixed** | **0.591(0.445-0.784)** | **14.5%** | **0.310** | **<0.001** | **BAA** | **Moderate** | **838** | **B** | **A** | **A** | **No** | **No** | **No** | **No** | **1** | **0.252** | **No** | **0.024** | **Strong** |
|  |  |  |  |  |  | **Recessive** |  | **fixed** | **0.784(0.591-1.040)** | **52.0%** | **0.125** | **0.091** |  |  |  |  |  |  |  |  |  |  |  |  |  |  |  |
|  |  | **G>C** | **Asian** | **1** | **210/420** | **Allelic** | **0.617** | **fixed** | **0.718(0.566-0.910)** | **Na** | **Na** | **0.006** |  |  |  |  |  |  |  |  |  |  |  |  |  |  |  |
|  |  |  |  |  |  | **Dominant** |  | **fixed** | **0.674(0.440-1.032)** | **Na** | **Na** | **0.07** |  |  |  |  |  |  |  |  |  |  |  |  |  |  |  |
|  |  |  |  |  |  | **Recessive** |  | **fixed** | **0.626(0.437-0.898)** | **Na** | **Na** | **0.011** |  |  |  |  |  |  |  |  |  |  |  |  |  |  |  |
|  |  | **G>C** | **Caucasian** | **2** | **305/324** | **Allelic** | **0.342** | **fixed** | **0.764(0.584-0.999)** | **0.0%** | **0.503** | **0.049** | **BAA** | **Moderate** | **427** | **B** | **A** | **A** | **No** | **NA** | **No** | **NA** | **1** | **NA** | **No** | **0.526** | **Weak** |
|  |  |  |  |  |  | **Dominant** |  | **fixed** | **0.534(0.364-0.783)** | **47.4%** | **0.168** | **0.001** | **BBA** | **Moderate** | **317** | **B** | **B** | **A** | **No** | **NA** | **No** | **NA** | **1** | **NA** | **No** | **0.163** | **Moderate** |
|  |  |  |  |  |  | **Recessive** |  | **fixed** | **1.158(0.725-1.849)** | **0** | **0.931** | **0.541** |  |  |  |  |  |  |  |  |  |  |  |  |  |  |  |
| **TP53** | **rs9895829** | **A>G** | **Overall** | **2** | **314/494** | **Allelic** | **0.078** | **fixed** | **0.982(0.664-1.455)** | **0** | **0.491** | **0.930** |  |  |  |  |  |  |  |  |  |  |  |  |  |  |  |
|  |  |  |  |  |  | **Dominant** |  | **fixed** | **0.981(0.651-1.478)** | **0** | **0.481** | **0.926** |  |  |  |  |  |  |  |  |  |  |  |  |  |  |  |
|  |  |  |  |  |  | **Recessive** |  | **fixed** | **Na** | **Na** | **Na** | **Na** |  |  |  |  |  |  |  |  |  |  |  |  |  |  |  |
|  |  |  | **Asian** | **1** | **210/420** | **Allelic** | **0.083** | **fixed** | **1.037(0.681-1.578)** | **Na** | **Na** | **0.867** |  |  |  |  |  |  |  |  |  |  |  |  |  |  |  |
|  |  |  |  |  |  | **Dominant** |  | **fixed** | **1.040(0.669-1.618)** | **Na** | **Na** | **0.860** |  |  |  |  |  |  |  |  |  |  |  |  |  |  |  |
|  |  |  |  |  |  | **Recessive** |  | **fixed** | **Na** | **Na** | **Na** | **Na** |  |  |  |  |  |  |  |  |  |  |  |  |  |  |  |
|  |  |  | **Caucasian** | **1** | **104/74** | **Allelic** | **0.049** | **fixed** | **0.692(0.237-2.019)** | **Na** | **Na** | **0.501** |  |  |  |  |  |  |  |  |  |  |  |  |  |  |  |
|  |  |  |  |  |  | **Dominant** |  | **fixed** | **0.681(0.228-2.035)** | **Na** | **Na** | **0.491** |  |  |  |  |  |  |  |  |  |  |  |  |  |  |  |
|  |  |  |  |  |  | **Recessive** |  | **fixed** | **Na** | **Na** | **Na** | **Na** |  |  |  |  |  |  |  |  |  |  |  |  |  |  |  |
| **VEGF** | **rs10434** | **A>G** | **Asian** | **6** | **1167/1524** | **Allelic** | **0.635** | **fixed** | **0.909(0.812-1.018)** | **0** | **0.974** | **0.098** |  |  |  |  |  |  |  |  |  |  |  |  |  |  |  |
|  |  |  |  |  |  | **Dominant** |  | **fixed** | **0.851(0.685-1.058)** | **0** | **0.995** | **0.146** |  |  |  |  |  |  |  |  |  |  |  |  |  |  |  |
|  |  |  |  |  |  | **Recessive** |  | **fixed** | **0.902(0.768-1.058)** | **0** | **0.965** | **0.206** |  |  |  |  |  |  |  |  |  |  |  |  |  |  |  |
|  | **rs1570360** | **A>G** | **Asian** | **3** | **527/692** | **Allelic** | **0.254** | **fixed** | **1.229(1.025-1.475)** | **0** | **0.774** | **0.026** | **BAC** | **Weak** | **658** | **B** | **A** | **C** | **First study** | **Yes** | **No** | **NA** | **0.296** | **0.269** | **No** | **0.341** | **Weak** |
|  |  |  |  |  |  | **Dominant** |  | **fixed** | **1.230(0.975-1.552)** | **0** | **0.816** | **0.081** |  |  |  |  |  |  |  |  |  |  |  |  |  |  |  |
|  |  |  |  |  |  | **Recessive** |  | **fixed** | **1.358(0.949-1.945)** | **0** | **0.995** | **0.094** |  |  |  |  |  |  |  |  |  |  |  |  |  |  |  |
|  | **rs2010963** | **C>G** | **Asian** | **7** | **1489/1867** | **Allelic** | **0.338** | **random** | **1.249(1.089-1.432)** | **46.4%** | **0.083** | **0.001** | **ABA** | **Moderate** | **3179** | **A** | **B** | **A** | **No** | **No** | **No** | **None** | **0.368** | **0.681** | **No** | **0.027** | **Strong** |
|  |  |  |  |  |  | **Dominant** |  | **fixed** | **1.393(1.190-1.630)** | **0** | **0.504** | **<0.001** | **AAA** | **Strong** | **2399** | **A** | **A** | **A** | **No** | **No** | **No** | **None** | **0.368** | **0.279** | **No** | **0.001** | **Strong** |
|  |  |  |  |  |  | **Recessive** |  | **fixed** | **1.294(1.098-1.524)** | **34.8%** | **0.163** | **0.002** | **BBA** | **Moderate** | **780** | **B** | **B** | **A** | **No** | **No** | **No** | **None** | **1** | **0.701** | **No** | **0.038** | **Strong** |
|  | **rs3025039** | **C>T** | **Asian** | **8** | **1671/2049** | **Allelic** | **0.230** | **fixed** | **1.248(1.120-1.391)** | **0** | **0.941** | **<0.001** | **AAA** | **Strong** | **1824** | **A** | **A** | **A** | **No** | **No** | **No** | **No** | **0.174** | **0.195** | **No** | **0.001** | **Strong** |
|  |  |  |  |  |  | **Dominant** |  | **fixed** | **1.222(1.066-1.399)** | **0** | **0.997** | **0.004** | **AAC** | **Weak** | **1523** | **A** | **A** | **C** | **Publication bias, small-study** | **No** | **No** | **No** | **0.035** | **0.054** | **No** | **0.065** | **Weak** |
|  |  |  |  |  |  | **Recessive** |  | **fixed** | **1.596(1.253-2.032)** | **0** | **0.702** | **<0.001** | **BAA** | **Moderate** | **301** | **B** | **A** | **A** | **No** | **No** | **No** | **No** | **0.174** | **0.253** | **No** | **0.009** | **Strong** |
|  | **rs699947** | **A>C** | **Asian** | **4** | **709/874** | **Allelic** | **0.679** | **fixed** | **0.713(0.615-0.827)** | **0** | **0.593** | **<0.001** | **AAA** | **Strong** | **2037** | **A** | **A** | **A** | **No** | **No** | **No** | **None** | **0.308** | **0.957** | **No** | **0.000** | **Strong** |
|  |  |  |  |  |  | **Dominant** |  | **fixed** | **0.611(0.462-0.810)** | **0** | **0.776** | **0.001** | **AAA** | **Strong** | **1346** | **A** | **A** | **A** | **No** | **No** | **No** | **None** | **0.734** | **0.990** | **No** | **0.041** | **Strong** |
|  |  |  |  |  |  | **Recessive** |  | **fixed** | **0.685(0.559-0.840)** | **0** | **0.687** | **<0.001** | **BAA** | **Moderate** | **691** | **B** | **A** | **A** | **No** | **No** | **No** | **None** | **0.308** | **0.697** | **No** | **0.009** | **Strong** |
|  | **rs833061** | **C>T** | **Asian** | **2** | **358/358** | **Allelic** | **0.624** | **fixed** | **0.788(0.638-0.974)** | **34.6%** | **0.216** | **0.027** | **BBA** | **Moderate** | **853** | **B** | **B** | **A** | **No** | **NA** | **No** | **NA** | **1** | **NA** | **No** | **0.358** | **Weak** |
|  |  |  |  |  |  | **Dominant** |  | **fixed** | **0.714(0.477-1.070)** | **14.6%** | **0.279** | **0.103** |  |  |  |  |  |  |  |  |  |  |  |  |  |  |  |
|  |  |  |  |  |  | **Recessive** |  | **fixed** | **0.735(0.540-1.000)** | **0** | **0.333** | **0.05** |  |  |  |  |  |  |  |  |  |  |  |  |  |  |  |
| **VMP1** | **rs1295925** | **T>C** | **Asian** | **2** | **1300/1300** | **Allelic** | **0.450** | **fixed** | **0.847(0.759-0.945)** | **0** | **0.597** | **0.003** | **AAA** | **Strong** | **2236** | **A** | **A** | **A** | **No** | **NA** | **No** | **NA** | **1** | **NA** | **No** | **0.053** | **Strong** |
|  |  |  |  |  |  | **Dominant** |  | **fixed** | **0.767(0.651-0.902)** | **0** | **0.646** | **0.001** | **AAA** | **Strong** | **1725** | **A** | **A** | **A** | **No** | **NA** | **No** | **NA** | **1** | **NA** | **No** | **0.026** | **Strong** |
|  |  |  |  |  |  | **Recessive** |  | **fixed** | **0.868(0.715-1.054)** | **0** | **0.711** | **0.153** |  |  |  |  |  |  |  |  |  |  |  |  |  |  |  |
| **XRCC1** | **rs1799782** | **G>A** | **Asian** | **2** | **318/523** | **Allelic** | **0.202** | **fixed** | **1.102(0.862-1.407)** | **0** | **0.362** | **0.438** |  |  |  |  |  |  |  |  |  |  |  |  |  |  |  |
|  |  |  |  |  |  | **Dominant** |  | **fixed** | **1.099(0.816-1.479)** | **0** | **0.578** | **0.535** |  |  |  |  |  |  |  |  |  |  |  |  |  |  |  |
|  |  |  |  |  |  | **Recessive** |  | **fixed** | **1.228(0.667-2.262)** | **90.0%** | **0.295** | **0.510** |  |  |  |  |  |  |  |  |  |  |  |  |  |  |  |
|  | **rs25487** | **T>C** | **Asian** | **2** | **318/523** | **Allelic** | **0.481** | **fixed** | **1.405(1.132-1.745)** | **0** | **0.433** | **0.002** | **BAA** | **Moderate** | **854** | **B** | **A** | **A** | **No** | **NA** | **No** | **NA** | **1** | **NA** | **No** | **0.052** | **Moderate** |
|  |  |  |  |  |  | **Dominant** |  | **fixed** | **1.488(1.055-2.099)** | **0** | **0.902** | **0.024** | **BAA** | **Moderate** | **597** | **B** | **A** | **A** | **No** | **NA** | **No** | **NA** | **1** | **NA** | **No** | **0.463** | **Weak** |
|  |  |  |  |  |  | **Recessive** |  | **fixed** | **1.564(1.114-2.195)** | **50.1%** | **0.157** | **0.010** | **BCA** | **Weak** | **257** | **B** | **C** | **A** | **No** | **NA** | **No** | **NA** | **1** | **NA** | **No** | **0.313** | **Weak** |
|  | **rs25489** | **C>T** | **Asian** | **2** | **318/523** | **Allelic** | **0.073** | **fixed** | **1.227(0.856-1.758)** | **0** | **0.703** | **0.265** |  |  |  |  |  |  |  |  |  |  |  |  |  |  |  |
|  |  |  |  |  |  | **Dominant** |  | **fixed** | **1.211(0.820-1.789)** | **0** | **0.890** | **0.336** |  |  |  |  |  |  |  |  |  |  |  |  |  |  |  |
|  |  |  |  |  |  | **Recessive** |  | **fixed** | **1.653(0.474-5.767)** | **0** | **0.515** | **0.430** |  |  |  |  |  |  |  |  |  |  |  |  |  |  |  |
| **XRCC3** | **rs861539** | **G>A** | **Asian** | **2** | **288/440** | **Allelic** | **0.272** | **fixed** | **1.572(1.252-1.975)** | **0** | **0.882** | **<0.001** | **BAA** | **Moderate** | **455** | **B** | **A** | **A** | **No** | **NA** | **No** | **NA** | **1** | **NA** | **No** | **0.006** | **Strong** |
|  |  |  |  |  |  | **Dominant** |  | **fixed** | **1.573(1.161-2.133)** | **0** | **0.902** | **0.003** | **BAA** | **Moderate** | **371** | **B** | **A** | **A** | **No** | **NA** | **No** | **NA** | **1** | **NA** | **No** | **0.151** | **Moderate** |
|  |  |  |  |  |  | **Recessive** |  | **fixed** | **2.230(1.395-3.566)** | **0** | **0.896** | **0.001** | **CAA** | **Weak** | **84** | **C** | **A** | **A** | **No** | **NA** | **No** | **NA** | **1** | **NA** | **No** | **0.240** | **Weak** |

Note: A, adenine; C, cytosine; G, guanine; T, thymine; OR, odds ratio; CI, confidence interval; MAF, minor allelic frequency in control; NA, not applicable; FPRP, false positive report probability.

^a^Allelic: Minor allelic (bold) versus major allelic (reference).

^b^Venice criteria grades are for amount of evidence, replication of the association and protection from bias.

^c^The prior probability of FPRP is 0.05, and the FPRP level of noteworthiness is 0.20.
